# Estimating animal population density using passive acoustics

**DOI:** 10.1111/brv.12001

**Published:** 2012-11-29

**Authors:** Tiago A Marques, Len Thomas, Stephen W Martin, David K Mellinger, Jessica A Ward, David J Moretti, Danielle Harris, Peter L Tyack

**Affiliations:** 1 Centre for Research into Ecological and Environmental Modelling, University of St AndrewsThe Observatory, Buchanan Gardens, Fife, KY16 9LZ, UK; 2 Centro de Estatística e Aplicações da Universidade de LisboaBloco C6, Piso 4, 1749-016, Lisboa, Portugal; 3 Space and Naval Warfare Systems Center Pacific53560 Hull Street, San Diego, CA, 92152, USA; 4 Cooperative Institute for Marine Resources Studies, Oregon State University and NOAA Pacific Marine Environmental Laboratory2030 SE Marine Science Drive, Newport, OR, 97365, USA; 5 Naval Undersea Warfare Center1176 Howell Street, Newport, RI, 02841, USA; 6 Sea Mammal Research Unit, Scottish Oceans Institute, University of St. AndrewsFife, KY16 8LB, UK

**Keywords:** acoustic surveys, bioacoustics, density estimation, distance sampling, passive acoustic monitoring, spatially explicit capture-recapture, fixed sensors, hydrophones

## Abstract

Reliable estimation of the size or density of wild animal populations is very important for effective wildlife management, conservation and ecology. Currently, the most widely used methods for obtaining such estimates involve either sighting animals from transect lines or some form of capture-recapture on marked or uniquely identifiable individuals. However, many species are difficult to sight, and cannot be easily marked or recaptured. Some of these species produce readily identifiable sounds, providing an opportunity to use passive acoustic data to estimate animal density. In addition, even for species for which other visually based methods are feasible, passive acoustic methods offer the potential for greater detection ranges in some environments (e.g. underwater or in dense forest), and hence potentially better precision. Automated data collection means that surveys can take place at times and in places where it would be too expensive or dangerous to send human observers.

Here, we present an overview of animal density estimation using passive acoustic data, a relatively new and fast-developing field. We review the types of data and methodological approaches currently available to researchers and we provide a framework for acoustics-based density estimation, illustrated with examples from real-world case studies. We mention moving sensor platforms (e.g. towed acoustics), but then focus on methods involving sensors at fixed locations, particularly hydrophones to survey marine mammals, as acoustic-based density estimation research to date has been concentrated in this area. Primary among these are methods based on distance sampling and spatially explicit capture-recapture. The methods are also applicable to other aquatic and terrestrial sound-producing taxa.

We conclude that, despite being in its infancy, density estimation based on passive acoustic data likely will become an important method for surveying a number of diverse taxa, such as sea mammals, fish, birds, amphibians, and insects, especially in situations where inferences are required over long periods of time. There is considerable work ahead, with several potentially fruitful research areas, including the development of (*i*) hardware and software for data acquisition, (*ii*) efficient, calibrated, automated detection and classification systems, and (*iii*) statistical approaches optimized for this application. Further, survey design will need to be developed, and research is needed on the acoustic behaviour of target species. Fundamental research on vocalization rates and group sizes, and the relation between these and other factors such as season or behaviour state, is critical. Evaluation of the methods under known density scenarios will be important for empirically validating the approaches presented here.

## CONTENTS

- Introduction 288
  1. Why passive acoustics? 288
  2. Canonical density estimator 289
  3. Historical perspective 290
- Background material 291
  1. Collection and analysis of passive acoustic data 291
    - Data collection 291
    - Sound analysis 291
  2. Overview of existing methods to estimate animal abundance 292
    - Census and plot sampling 292
    - Distance sampling 293
    - Mark-recapture 294
    - Spatially explicit capture recapture 294
    - Other model-based approaches 295
    - Variance estimation 295
    - Analytic variance estimation 295
    - Bootstrap variance estimation 296
- Framework for estimating density from acoustic data 296
  1. Active acoustic surveys 296
  2. Type of objects detected acoustically 297
  3. Towed acoustic sensors 298
- Estimating density using fixed passive acoustics: examples and case studies 299
  1. Census/strip transects 299
  2. Distance sampling 299
  3. Detection function estimated from auxiliary data 300
    - Trials using sounds for which location is known 300
    - Acoustic modelling 301
  4. Mark-recapture 301
  5. Mark-recapture distance sampling 301
  6. Spatially explicit capture-recapture 301
- Discussion 302
  1. Calibration studies and other approaches 302
  2. Accuracy of density estimates 303
  3. Getting multipliers right 303
  4. Future research areas 304
- VI. Conclusions 305
- VII. Acknowledgements 306
- VIII. References 306

## I. INTRODUCTION

The management and conservation of wildlife are increasingly important concerns in a world of limited resources and increasing human population, with many wild populations under pressure from anthropogenic activities. To develop effective management and conservation for a given species, the most basic questions that often must be addressed first are ‘how many are there?’ and ‘is the population increasing or decreasing?’. For example, the International Union for Conservation of Nature criteria for defining conservation status depend heavily on population sizes (IUCN, 2003). Although simple questions, the answers are often difficult, laborious and costly to obtain. Therefore, developing and promoting the use of reliable and effective methods to estimate abundance is essential to implement management and conservation policies. Here, we focus on the nascent science of passive acoustic density estimation: methods to estimate population density or abundance based on detecting sounds naturally produced by animals. Acoustic density estimation must not be seen as a panacea; however, as we show here, it is a fast-developing field with enormous potential.

### (1) Why passive acoustics?

Wildlife abundance estimation is dominated by methods based on two modes of data acquisition: visual observations and physical capture (trapping). Probably the most common survey method is visually based distance sampling (Buckland *et al*., 2001), the required data being the distances to animals sighted from a set of random transect lines or points. Alternatively, if sighted animals have unique markings, then mark-recapture (MR, also called capture-recapture; Williams, Nichols & Conroy, 2002) methods can be used, the data being capture histories representing, for a set of survey occasions, when each animal was seen. Trapping is also commonly used to generate MR data: animals may be trapped once, given unique markings, and re-sighted or re-trapped on subsequent occasions.

Passive acoustics offers an alternative survey mode in situations where visual surveys or physical trapping are difficult, expensive, or dangerous. Many species are visually cryptic: for example, they may be small, camouflaged, nocturnal, or hidden underground, or they may live under water or in thick foliage where they are difficult to sight. Some species are not amenable to trapping due to lack of effective methods (particularly for recapture in traps) or welfare concerns. However, many species produce readily detectable and distinguishable sounds that may be used to estimate abundance and density.

Even if visual or trap-based methods are possible, passive acoustic methods may be preferable. First, animals that produce loud or frequent sounds may be detectable at greater ranges acoustically than by other means. Second, unlike (most) visual surveys, passive acoustic surveys can operate under any light conditions (e.g. both day and night, or in fog), being less affected by weather conditions. Third, passive acoustics is highly amenable to automated data collection and processing, so large amounts of data can readily be analysed. By contrast, visual surveys are largely performed by human observers (although this is changing as digital imagery technology improves), and trapping studies are also usually labour-intensive. It is often easier to quantify factors affecting probability of detection by automated systems than by human observers. Lastly, automated data collection means that information can be gathered in environments where it is not easy for human observers to work (e.g. deep or polar oceans).

Although passive acoustic density estimation may potentially be applied to a wide variety of terrestrial and aquatic taxa, cetaceans have been so far the focus of most exclusively acoustic-based applications (although we note that many bird surveys are mostly, but rarely exclusively, based on acoustic data). Why? First, sound propagation is more efficient in water than in air, while energy from light is absorbed more than that from sound as it passes through water; hence many aquatic species rely on acoustics for communication. Second, visual surveys are effective for many cetaceans, but can be very expensive, usually requiring large investments of ship time and teams of trained observers. Third, several deep-diving cetacean species forage at depths where light does not penetrate and hence use echolocation for foraging, making them ideal subjects for passive acoustics.

There have been a number of reviews of the potential for acoustic monitoring, although none has described methods for density estimation. Indeed, until very recently, this was not thought feasible (e.g. Wood, 2010). Cato *et al*. (2006) review the progress and challenges for passive acoustic monitoring of cetaceans. Mellinger *et al*. (2007) review the use of acoustic methods to gather information about cetacean species, listing abundance estimation as a key area of future development. Gannon (2008) reviews passive acoustics in fisheries. Van Parijs *et al*. (2009) present an overview of research and management applications of passive acoustics at sea. Blumstein *et al*. (2011) assess the potential of acoustic monitoring in terrestrial habitats, noting its possible application for abundance estimation. It is our hope that the present review might act as a catalyst, promoting the use of passive acoustic methods in terrestrial environments, where currently the approach seems to be underutilized.

### (2) Canonical density estimator

Suppose that a wildlife survey takes place within some defined study area of size *A*. A large number of sample plots, with total area *a*, are located at random (a systematic random design is usually best, Borchers, Buckland & Zucchini, 2002), and *n* animals are counted. Assuming all animals within the sample plots are counted, then density, *D*, is estimated as


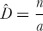
(1)

and the estimated abundance, or population size, is simply density times the size of the study area: 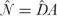 (circumflexes denote that a quantity is estimated rather than known). In most cases, however, we need to account for some animals not being detected within the sample plots. If we can estimate the probability *p* of detecting an animal within the sample plots, density can be estimated as


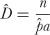
(2)

Another way to view the effect of missing animals within sample plots is that it effectively reduces the size of the area sampled. This is a helpful way to think about some of the methods – by defining the effective area of detection, *a_e_*, where the number of animals within this area is on average the same as the number of detected animals within *a* (Buckland *et al*., 2001, p. 54). Note that, naturally from this definition, *a_e_* = 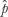
*a*.

In passive acoustic surveys, it is often not possible to count the number of animals directly – for example, we may be able to count individual vocalizations but are unsure of how many animals produced them. Using the number of vocalizations in the above formula produces an estimate of the vocalization density; this can then be divided by an estimate of vocalization rate to produce an estimate of animal density. If we can isolate and count the number of vocalizing groups, we then can estimate the density of animals by multiplying the number of groups by an estimate of average group size and dividing by the probability of a group vocalizing during the survey period. In general, the factors that convert an indirect estimate into an animal density estimate are called ‘multipliers’. Another common multiplier is used to account for ‘false positive’ detections – instances where a sound is classified as coming from the species of interest, but was actually something else. Hence, a canonical density estimator for passive acoustic surveys can be written:


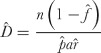
(3)

where *n* is the number of detected ‘objects’ (vocalizations, groups, etc.), *f* is the proportion of detections that are false positives, *p* is the probability of detecting an object within the area *a*, and *r* represents the multiplier(s) that converts object density to animal density. While false positives receive considerable and certainly well-deserved attention in the context of acoustic surveys (e.g. Marques *et al*., 2009, 2012; McClintock *et al*., 2010), the same problem potentially occurs in visual surveys and is often ignored. The detector performance, in terms of false negatives and false positives, must be accounted for otherwise density estimates will be biased. This performance might be far from ideal and yet density estimation still be possible (e.g. Marques *et al*., 2009), provided the operating characteristics of the system are rigorously assessed.

Excluding very specific settings outside this framework, using passive acoustics to estimate density involves: (*i*) identifying sounds to use that relate to animal density; (*ii*) collecting a sample of sounds, *n*, using a well-designed survey protocol; (*iii*) estimating the false positive rate, *f*; (*iv*) determining the probability of detection, *p*; (*v*) obtaining an estimate of the multiplier *r* that translates sound density to animal density. In later sections we detail approaches for estimating these quantities, and give examples. We note at the outset (and discuss further later) that the most reliable estimates of *f*, *p* and *r* will come from data collected at the same time and region as *n*, as opposed to extrapolation from other surveys, or modelled values. However, extrapolation or modelling is often required in the absence of better information; in such cases, assumptions and caveats should be understood, respected and discussed.

### (3) Historical perspective

Many underwater sounds were recorded and described long before they were definitively attributed to specific animal species. A classic example is the ‘boing’ sound (Wentz, 1964), only recently attributed to minke whales *Balaenoptera acutorostrata* (Rankin & Barlow, 2005). Many species produce sound, either actively with a purpose (e.g. sexual display, echolocation) or as a consequence of their activity (e.g. breathing, feeding, or locomotion). A wide variety of animal taxa produce sound, including insects, amphibians, fish, birds and mammals. For example, more than 800 species of fish have been identified as producing sound, and hence potentially are acoustically detectable (Juanes, 2002). Many economically important species such as cod (*Gadus morhua*), herring (*Clupea harengus*) and haddock (*Melanogrammus aeglefinus*) produce sound (Juanes, 2002; Luczkovich, Mann & Rountree, 2008). Most of these are species-specific low-frequency sounds, which propagate over relatively long distances (Luczkovich *et al*., 2008). Sounds can be used in a wide range of applications to obtain important information about the animals that produce them (e.g. Gordon, 1991; Russo & Jones, 2003; Zhang *et al*., 2003; Holt, 2008). Sound has also been used to study group size (e.g. Van Parijs, Smith & Corkeron, 2002; Payne, Thompson & Kramer, 2003). George *et al*. (2004) presented an example in which acoustic detections were used to estimate the proportion of whales available for detection by visual observers, and hence to better estimate abundance from the visual observations. Localization of acoustic signals has also been used to direct visual observers towards animals (e.g. Falcone *et al*., 2009). Sound is often the primary cue used for initial detection of birds (e.g. Alldredge, Simons & Pollock, 2007), primates (e.g. Defler & Pintor, 1985), bats (e.g. O'Farrell & Gannon, 1999) and amphibians (Driscoll, 1998).

The idea that acoustic data might contain useful information about animal density has evolved over the last few decades. Passive acoustic monitoring (PAM) refers loosely to methods using sounds made by animals to make inferences about their distribution and occurrence over space and time. Zimmer (2011) introduces this field. Passive acoustics relies on the detection of sounds produced by the target (animal), whereas active acoustics relies on the detection of the return echo of a transmitted acoustic signal that reflects off the target. For a wide range of sound-producing taxa, acoustic information has been extensively used to make inferences about presence in a given area (e.g. O'Farrell & Gannon, 1999; Kimura *et al*., 2009; Bucci, Petryszyn & Krausman, 2010; Clark, Brown & Corkeron, 2010) or relative abundance (e.g. Forrest, 1988; Van Parijs, Hastie & Thompson, 1999; Ichikawa *et al*., 2006; Van Parijs & Clark, 2006; Oleson *et al*., 2007; Stafford *et al*., 2007; Širović *et al*., 2009). Recently, it has been recognized that acoustic data could be the primary source to derive absolute measures of abundance. While obvious after the fact, this is a recent development, because the data acquisition and processing of sound recordings is far more complicated than that based on visual data and because the vocal behaviour of a species must be well characterized before acoustics can be used for reliable density estimation.

Many coordinated research efforts have been developed in recent years to monitor animal populations acoustically, including the 2002 workshops on passive acoustics in fisheries (Juanes, 2002) and marine mammals (Mellinger & Barlow, 2003), and the 2009 workshop ‘Status and Applications of Acoustic Mitigation and Monitoring Systems for Marine Mammals’ (Bingham, 2011). Similar efforts focused specifically on cetacean density estimation. A 2009 symposium on ‘Estimating Cetacean Density from Passive Acoustics’ was held at Scripps Institute of Oceanography and a dedicated session was held at the 159th meeting of the Acoustical Society of America. Since 2004 there have been dedicated biennial workshops on ‘Detection, Classification and Localization (DCL) of Marine Mammals using Passive Acoustics’, and the last two of these included a day dedicated to estimating cetacean abundance from passive acoustic data (Pavan, Adam & Thomas, 2010). These efforts illustrate the importance of acoustic methods for density estimates of cetaceans and fish, as well as other animal groups. Perhaps surprisingly, a recent Marine Ecology Progress Series Theme Section on acoustics in marine ecology (Southall & Nowacek, 2009) had no contribution focused on density estimation. Given the above, we believe that this is a timely review: tools are available, and the first steps have been taken. Density estimation from passive acoustics is an entire sub-discipline waiting to be explored and developed further.

## II. BACKGROUND MATERIAL

### (1) Collection and analysis of passive acoustic data

The collection and analysis of acoustic data has a number of peculiarities that are relevant for density estimation, and we review these briefly here.

#### (a) Data collection

Data for density estimation from passive acoustic sensors can be collected in terrestrial and marine environments using a variety of platforms, including fixed and mobile options.

Fixed passive acoustic systems are described only briefly here; see Mellinger *et al*. (2007) for details. Autonomous recorders comprise the most common fixed sensor platform, having been developed by navies, academic laboratories, and private companies (Fox, Matsumoto & Lau, 2001; Clark, Borsani & Notarbartolo-di-Sciara, 2002; Wiggins, 2003; Multi-Électronique, 2011; Wildlife Acoustics Inc., 2012). These devices are anchored to the sea floor and record ambient sound either continuously or with an on-off sampling schedule (which may be adaptive, depending on what was detected previously). Important considerations for choosing one of these systems include frequency band, sensitivity and dynamic range, maximum deployment duration, robustness/durability, maximum deployment depth (for marine devices), initial purchase, deployment, recovery and refurbishment costs, and difficulty of recovery (Dudzinski *et al*., 2011). Some devices have an on-board processing capability, and record only summary statistics or full bandwidth recordings when signals of interest are detected, allowing longer deployment times for a given size of instrument, but hindering analysis of ancillary acoustic data.

Widely spaced microphone or hydrophone systems can transmit acoustic signals to data-recording equipment using wireless links or cables. In terrestrial habitats, such systems are typically used over relatively small distances – tens to hundreds of meters – since large cable lengths are relatively expensive compared to multiple independent autonomous recorders. In marine environments, large-area cabled systems are used where long-term monitoring is required and the operational cost of a portable system exceeds the installation cost of a fixed system. Navies have long used cabled acoustic arrays, and some of these have been made available to research biologists (e.g. Clark, 1995; Stafford *et al*., 2009). In recent years, both governments (e.g. Barnes *et al*., 2008) and private citizens (Veirs & Veirs, 2005) have installed cabled acoustic arrays for scientific use.

Another popular system in marine environments is the mobile hydrophone array. Such arrays can have anywhere from two to hundreds of hydrophones, and are typically towed behind a vessel on a cable tens to thousands of meters long. Arrays typically permit localization of animal sound sources up to several times the aperture of the array, a key step in estimating population density as described below. Short-baseline (centimetres to metres) arrays typically permit instantaneous estimation of only the bearing to vocalizing animals, information which can nevertheless be useful for estimating the number of dispersed animals calling simultaneously. Successive bearings can also be calculated over time as a vessel moves, and then used to estimate positions of animals that move slowly relative to the vessel. Mobile hydrophones and hydrophone arrays are also being deployed on a variety of platforms based on newer technologies, including deep ocean gliders (Moore *et al*., 2007; Baumgartner *et al*., 2008) and autonomous wave-powered vessels (Willcox, Manley & Wiggins, 2009).

Animal-borne recording tags (Burgess *et al*., 1998; Johnson & Tyack, 2003) provide another means of collecting passive acoustic data. Such tags, which record sound while attached to an animal and often document animal movement, can be used to estimate the sound production rate of animals in a population. In addition, such tags are critical for documenting a species' vocal behaviour and its relationship to physical behaviour. For example, based on data from a tag that collects both acoustic and kinematic data (the DTAG; Johnson & Tyack, 2003), *Ziphius cavirostris* and *Mesoplodon densirostris* beaked whale species are known only to produce sounds during deep foraging dives, at depths below 200 m (Tyack *et al*., 2006). When combined with other microphones or hydrophones, tags can also be used to estimate the detection function – the probability of detecting sounds as a function of distance (see Section IV.3*a*).

#### (b) Sound analysis

Real-time or recorded acoustic data can be manually analysed using software that displays the data, typically as a spectrogram. An operator inspects the data, often both visually and aurally, and finds sounds made by the target species. This method is labour-intensive, but often necessary for species that are poorly known and/or difficult to detect and classify automatically.

An alternative is automated analysis, in which a software system is used to isolate the sounds of interest. This is usually less costly and, given that it is repeatable and objective, more amenable to performance quantification. Such systems use detection and classification algorithms to detect and distinguish the target calls from background noise and interfering sounds. Many such algorithms have been developed for a wide variety of animal sounds (e.g. Mellinger & Clark, 2000; Brandes, Naskrecki & Figueroa, 2006; Abbot, Premus & Abbot, 2010; Bardeli *et al*., 2010). Important factors in choosing an algorithm include the acoustic structure of the signal, the amount of variation in the species' sounds (relatively stereotyped or more variable), the nature of the background noise and interfering sounds (similarity to the target sounds), whether detection/classification parameters are already available for the target sound, and if not, the difficulty of training the detection/classification method for the target sound (some methods require a handful of examples for training, some require hundreds or thousands). Sound types can be described as tonal (moans, whistles, tones, etc.), impulsive (short-duration clicks, impulses, etc.), roar- or hiss-like (longer-duration broadband sounds), or combinations of these. Frequencies can vary from the infrasonic (< 20 Hz) to ultrasonic (> 20 kHz) and the sound duration may go from tens of microseconds to tens of seconds. Popular software packages for automated detection and classification include ISHMAEL (Mellinger, 2001), PAMGUARD (Gillespie *et al*., 2008), and XBAT (Figueroa, 2011).

A key point to consider is that any method for the detection and classification of sounds will produce false negatives (some sounds of interest are missed, either not detected or incorrectly classified), and false positives (detections are registered in the absence of the sounds of interest). For the development of robust passive acoustic density estimation algorithms, these parameters should be quantified in the environment of interest since they often depend on the density of competing sound sources such as other species.

Another relevant aspect of sound analysis in the context of animal abundance estimation, and an area of active research, is the ability to localize the sound source. This is typically done utilizing the time difference of arrival of the same sound at multiple widely spaced fixed sensors (e.g. Bower & Clark, 2005; Širović, Hildebrand & Wiggins, 2007; Ward *et al*., 2008; Parsons *et al*., 2009). Four closely spaced sensors can provide a bearing to the sound source, and when combined with information on the species, such as depth when vocalizing, localization is also possible (e.g. Wiggins, McDonald & Hildebrand, 2012). Location information may also be obtained utilizing two or more directional sensors (McDonald, 2004), or towed sensors (e.g. Li *et al*., 2009). If the position of the animal within a time window can be assumed fixed, cross-bearings can be used to estimate the animal's position as the platform moves.

Even when the location of calling animals is not available, the distance to a calling whale from a sensor allows straightforward implementations of distance sampling (see below, section IV.2). A single sensor can provide the distance to a calling animal using propagation modelling techniques which range from single-sensor multipath arrivals (McDonald & Fox, 1999; Aubauer, Lammers & Au, 2000) to normal mode dispersion of whale calls (Munger, Wiggins & Hildebrand, 2011). When two or more sensors are available, techniques such as exploiting the received level differences and arrival times (Cato, 1998) also allow ranging. The received level of a sound may also provide (imprecise) information about the distance of the source, as may the spectral content, since high frequencies are absorbed more rapidly than low ones.

### (2) Overview of existing methods to estimate animal abundance

Estimation of animal abundance and density is an extensive field. The classic book by Seber (1982) and the general reviews by Seber (1986, 1992) and Schwarz & Seber (1999) provide details and extensive references about the different methods. Good overviews are given by Borchers *et al*. (2002) and Williams *et al*. (2002, Part III). Our goal here is to provide an outline of the key existing approaches from which density estimates might be obtained, laying the ground work for developing some of these in the context of acoustic data.

Animal abundance is traditionally estimated using methods based on visual observations. Therefore, methodological development has focussed on visually acquired data. Abundance and density estimation methods based on visual data build almost exclusively on one of two different inferential approaches: mark-recapture (MR) and distance sampling (DS). A recent development, spatially explicit capture-recapture (SECR), blends the two methods. Note that we use the terms ‘capture-recapture’ and ‘mark-recapture’ interchangeably; and also that the animals are often not strictly captured or recaptured (e.g. detection *via* camera traps, hair snares or acoustic sensors). The key for MR is the ability to recognize whether an animal has already been detected or whether the detection represents a first encounter.

#### (a) Census and plot sampling

Ideally, one would like to count all the animals in the target population, i.e., implement a census. However, situations in which this is possible are rare, and usually require small populations occupying restricted areas. Hence, to obtain abundance estimates, investigators must often rely on sampling.

Although a total count of the population is seldom possible, it might still be possible to perform a total count over some randomly chosen plots. This will allow density estimates for the survey area to be obtained using conventional sampling methods. These are often referred to as strip transects or plot sampling. However, these methods are often abused, being applied to situations where the key assumption, that all animals in the survey plots are detected, is false. This leads to an underestimation of density.

Plot sampling is usually a design-based approach: sampled plots are assumed to be a random sample of a larger number of plots, and hence the density estimated over these is valid for the wider survey area. The abundance over the entire survey area needs to account for the proportion of the area surveyed (assuming a simple random sampling scheme). Alternatively, one could consider a model-based approach, where inferences over the wider survey region are based on a model which relates abundance to covariates. Hence the distinction between a design-based and model-based approach is that in the former the known properties of the random design are used to link what was observed in the sample to the rest of the study area, while in the latter a model of animal distribution is used to make this link (e.g. regression approach in which density is predicted as a function of covariates).

#### (b) Distance sampling

The probability of detecting an animal typically depends on its distance from the observer or sensor. A statistical method called ‘distance sampling’ uses detection distances to estimate the area effectively searched, or equivalently the average probability of detection within some fixed truncation distance (Buckland *et al*., 2001, 2004). This is then used to correct the observed number of individuals, or groups, for those that went undetected. The methods rely on the random placement of a sufficiently large number of line or point transects over the area of interest. Typically a systematic design is used to enforce good coverage of the entire area. The distances to the detected animals are used to model a detection function. The detection function, *g*(*y*), represents the probability of detecting an animal, given that it is located at distance *y* from the transect. The distance *y* corresponds to a perpendicular or radial distance depending on whether line or point transects are used. It can be shown (see, e.g. Borchers & Burnham, 2004, pp. 16–17) that the average probability *p* of detecting an animal in the covered area is given by


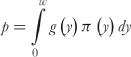
(4)

where *w* is a distance beyond which detections are ignored or assumed not to occur, usually referred to as the truncation distance, and π(*y*) is the distribution of distances to all animals, detected or not. Note this is an intuitive estimator, as it just represents the mean value of *g*(*y*) with respect to the available distances *y*. This *p* is then plugged in an estimator like the one presented in Equation (2).

A particular type of distance sampling is cue counting (Hiby & Ward, 1986), in which instead of detecting animals, one detects cues produced by them. This was originally developed for estimating whale density from whale blows, and is useful in general when it is possible to detect and count some cue (such as whale blows) but hard to determine which individual produced which cue. Instead, the density of cues is estimated (e.g. whale blows per unit area per unit time), and this is divided by an independently derived estimate of the average rate at which an animal produces cues (e.g. number of blows per unit time). In the original implementation, data on whale blows were collected along line transects, but only a radial sector ahead of the ship was surveyed, such that the methods are more closely related to point than line transects. Cue counting has also been applied to aural surveys of birds from points, where the cue is each individual call or song detected (e.g. Buckland, 2006). Again, independent information about cue rate (i.e. average number of calls or songs per unit time) is required to convert cue density to bird density.

Another approach applies when animals naturally occur in clusters, and the clusters become the object of analysis. Traditionally, the approach taken has been to obtain a density of clusters and then multiply that by an estimate of the mean cluster size in the population (see, e.g. Buckland *et al*., 2001, pp. 71–76). Larger clusters are often easier to detect than smaller ones, leading to a potential bias when determining population mean cluster size; this is often dealt with using multiple covariate distance sampling (MCDS, see Marques & Buckland, 2003; Marques *et al*., 2007).

Unbiased estimation from conventional distance sampling methods requires a number of assumptions, which we address in turn. Often overlooked, but strictly an assumption, the above formula is only useful because the distribution of animals *π*(*y*) is assumed to be known: uniform for line transects, and triangular for point transects. These distributions stem from the available area from the samplers as a function of distance, and are a direct consequence of transects being placed randomly within the study area; hence a safe assumption for proper survey designs. However in some poorly designed surveys, transects are placed along existing landscape features, like roads, rivers or shorelines. In this case, because the animals might also present a density gradient with respect to these features, the form of *π*(*y*) is unknown and might not be estimable from the conventional data. This leads to potentially severely biased estimates (e.g. Marques *et al*., 2010).

Additionally, we assume that: (*i*) animals on the line or at the point are detected with certainty, i.e. *g*(0) = 1; (*ii*) the animals do not move or the observation process is conceptually a snapshot, i.e. instantaneous in time; (*iii*) distances are measured without errors; and (*iv*) detections are statistically independent events. Methods are robust to the violation of some of these assumptions, in particular 4 (which generally only affects variance estimates). In the case of other assumptions, mild violation is unlikely to lead to serious problems, but moderate or severe violation can lead to considerable bias and should be avoided. Investigators should spare no effort to fulfil these assumptions at the study design and field methods level, rather than dealing with them at the analysis stage. We address the consequence of their failure in turn below. If a fraction of animals on the line are not detected [*g*(0)<1], then density estimates are proportionally low. This is the case both if observers fail to detect animals available for detection (perception bias), or if there is a fraction of the animals not available to be detected, say submerged or underground (availability bias). To address this assumption failure, in particular for perception bias, mark-recapture distance sampling methods have been developed that allow the estimation of *g*(0) (Laake & Borchers, 2004).

Although strictly a snapshot means an ‘instant’ in time, a period of time of negligible length, in practice what is required is that the period is such that animal movement is negligible within the time interval. If observers move considerably faster than the animals themselves, then bias from this source can be safely ignored. However, for highly mobile animals and in particular for point transects (in which by definition the observer stands still), even random movement can lead to considerable overestimation of density. Perhaps even more important, severe bias might result from unobserved responsive movement, typically overestimation of density if animals are attracted to the observer, and underestimation of density if animals avoid the observer. This assumption has received less attention in the literature, likely because it is difficult to obtain information about movements of unobserved animals.

The consequence of measurement error in estimated distances is very similar to that of animal movement. Random errors will typically lead to an overestimation of density (Marques, 2004), while underestimation and overestimation of distances will lead respectively to overestimation and underestimation of density. Provided the measurement error process can be modelled, this bias can be corrected (e.g. Borchers *et al*., 2010).

The independence assumption is required to estimate the parameters of the detection function model by maximum likelihood, but density estimates are extremely robust to its failure. While variance estimates are more likely to be affected, the recommended procedures, using an empirical estimator for the variance, are also very robust to this assumption failure (e.g. Buckland *et al*., 2001).

#### (c) Mark-recapture

A conceptually different approach to abundance estimation is mark-recapture (MR). Chapter 6 in Borchers *et al*. (2002) presents an overview of simple MR. This method requires the ability to recognize individuals within the population being studied. Historically done by marking the animals in some way, increasingly other methods of individual recognition such as photographic identification and genetic markers are being used. In the context of acoustic surveys, individual vocalizations sufficiently distinct to allow individual recognition would be required. The fundamental concept underlying MR is intuitive. One collects a sample of *n* animals and marks them, hence an unknown fraction of animals, *n*/*N*, becomes marked. A second sample is drawn. Given random animal mixing between samples, the proportion of marked animals in the new sample *p* is an estimate of the proportion of marked animals in the population. Hence, an estimate of population size is given by 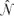
*=n/p*. This is called the Lincoln-Petersen estimator, but is rarely used nowadays. It has a number of unrealistic assumptions, namely that the population is closed (i.e. no deaths, births, immigration and emigration occur between capture occasions) and that all animals have the same probability of being captured (detected). When the latter is not true, estimates are biased low, and this is known as unmodelled heterogeneity in capture probabilities (see e.g. Link, 2003, for details and examples). Because not all animals have the same characteristics, some are more detectable than others. Hence, the sampled animals tend to be biased towards the more detectable animals, animal detection probability tends to be overestimated, and abundance underestimated. MR methods have evolved from purely closed population models to methods capable of dealing with open populations and incorporating multiple sources of heterogeneity in detection probabilities (hence reducing, but not really solving (Link, 2003), the issue of unmodelled heterogeneity). Nowadays MR methods are perhaps more commonly, and certainly less controversially, applied to obtain other relevant ecological parameters rather than abundance, such as survival.

Population size estimates derived from MR are not easily converted to density estimates, because the population being sampled is ill defined under most settings. The problem is that there is no rigorous way to assess the area that the sampling effectively covers. Hence, we have an estimate of *N*, but not the area it corresponds to (see e.g. Efford, 2004, for details). The use of conventional MR estimates for density estimation therefore tends to be a distant second choice, but is presented here because it provides a logical building block leading to the next method, spatially explicit capture recapture.

Note the close links between MR and DS; a combination of these two approaches, mark-recapture-distance-sampling (MRDS; for details and other references see Laake & Borchers, 2004), might help to address issues that neither of them can alone, by accounting simultaneously for availability bias and heterogeneity in detection probability (due to distance, and other relevant covariates).

#### (d) Spatially explicit capture recapture

The recent development of spatially explicit capture recapture (SECR; Efford, 2004; Borchers & Efford, 2008; Royle & Young, 2008) was motivated by two key issues in MR: (*i*) unmodelled heterogeneity in detected animals (i.e. not all animals have the same probability of being detected), and (*ii*) an ill-defined population (i.e. the surveyed area is defined *ad hoc* in MR). In SECR, the available information about the spatial location of the ‘captured’ animals (at the very least, the location of the ‘traps’ in which they are captured or detected) allows one to minimize issue (*i*) and resolve issue (*ii*) above. In the acoustic context, sounds are detected in multiple devices, rather than the same animal being detected over multiple ‘traps’. SECR combines both capture recapture and distance sampling models in a unifying framework (Borchers, 2012).

SECR was originally developed in the context of trapping studies of small mammals. A central concept is that of ‘home range’ centre: the home range does not need to have a biological meaning, and its centre is typically not observed. The probability of capturing an animal is modelled in terms of the distance from the traps to this unobserved location. This model is then used to obtain the detection probability associated with any given animal location. Because the home range centre for each individual is unobserved, it must be integrated out of the process. In layman's terms, it is equivalent to calculating an average detection probability, in which the average is with respect to all the positions where the animal's home range centre could be. The standard assumption is a uniform distribution in space, and the sum of the detection probabilities over space turns out to be the effective sampling area of a given set of traps, provided this assumption holds (Borchers, 2012). Part of the unmodelled heterogeneity in conventional MR often results from some animals being more likely to be detected because their home range centres are close to traps. The explicit inclusion of the location of the traps into the estimation procedure allows one to account for that component of heterogeneity. Further, the methods are based on a model for density that allows calculation of the effective sampled area for a given array of traps, and therefore density estimates can be obtained in a rigorous framework.

Different types of trap can be considered, and SECR methods have been applied to cage traps, hair snares, camera traps and acoustic detectors. Acoustic detectors are known as ‘proximity’ detectors: capture in one detector does not invalidate capture in any other detector (unlike a cage-trap, in which any one animal can only be trapped in one trap at each capture occasion). This opens the door to SECR estimates based on a single capture occasion (see Efford, Dawson & Borchers, 2009), which was impossible with conventional MR methods. The basic data for SECR are capture histories (i.e. vectors coding when and where each animal was captured). Hence it is required that animals, or their cues, can be individually recognizable.

#### (e) Other model-based approaches

Methods based on presence-absence (Royle & Nichols, 2003) or counts (Royle, 2004) of a given species over repeated visits to a given number of sites have also been used to estimate abundance. Again the idea is intuitively simple: the probability of detecting one or more individuals, i.e. of recording a site as being occupied, is directly related to the number of animals at the site. However, several strong assumptions are required: that the population is closed, detections are independent events and the individual detection probability is constant (across individuals, and over time). So, as with MR methods, these are inherently plagued by unmodelled heterogeneity in capture probability.

#### (f) Variance estimation

Often overlooked, precision measures for density estimates are as important as point estimates, because only then can one draw meaningful inferences from the reported values. The same point estimate for a given population, say 1000 individuals, will have very different meaning if the respective 95% confidence interval is (900, 1200) *versus* (50, 10000). Therefore, reliable estimates of precision must be obtained. A useful and often reported precision measure is the coefficient of variation (CV, the standard error of the estimate divided by the estimate), which provides a measure of precision independent of the scale of the measurement units.

There are two general approaches one can take to estimate the variance of some arbitrary estimator, which we present in turn below.

##### (i) Analytic variance estimation

The density and abundance estimators we consider in this section are the product of a number of random components (*r*_m_, *m* = 1,2,…,*M*) and constants (*q*_k_, *k* = 1,2,…,*K*), i.e. having a generic form


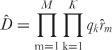
(5)

Typical constants relate to effort (e.g. recording time, number of sensors or line length) while the most obvious random component is the detection probability. Constants are manageable, as by definition they have no variance. If the variance in each of the random components can be quantified and the random components are independent, then


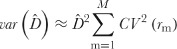
(6)

(this is an approximation based on the delta method; see e.g. Seber, 1982, p. 9). The key issue is estimating the required variance from each of the random components. For averages, weighted averages, or functions of maximum likelihood estimates this is usually straightforward. Note that if the random components are not independent, one needs to account for the correlation structure between these random components. Otherwise, the variance will be underestimated or overestimated, depending on the correlation structure (see Powell, 2007, equation 2, for the general case, and equation 15 for an example).

To obtain a confidence interval for density, the density estimator is often assumed to follow a log-normal distribution (e.g. Buckland *et al*., 2001, p. 77), leading to a (1-α)% confidence interval given by


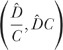
(7)

where


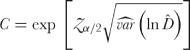
(8)

and


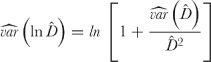
(9)

and z_α/2_ is the upper α/2 quantile of the standard Gaussian distribution. If some of the random multipliers are based on relatively small sample sizes then a *t*-distribution-based method can be used (Buckland *et al*., 2001, pp. 77–78).

##### (ii) Bootstrap variance estimation

A different approach is to use resampling strategies to estimate the variance (e.g. Manly, 2007). The non-parametric bootstrap is the approach used most often. The idea is to resample with replacement the independent sampling units (e.g. transects or sensors) to build a new ‘bootstrap’ dataset, and use this to obtain a new estimate of density. Repeating this procedure many times yields a set of density estimates. The empirical variance of those estimates approximates the variance of the original estimator. From this there are two approaches to obtain confidence intervals. Either one uses the estimated variance with the log-normal assumption as described above, or a percentile method, in which the (1-α)% confidence interval is given by the lowest and highest α/2 quantiles of the bootstrap estimates. Bootstrap offers a robust alternative to analytic variance and confidence interval equations, because of the mild distributional assumptions, and so is often recommended in practice (e.g. Buckland *et al*., 2001).

## III. FRAMEWORK FOR ESTIMATING DENSITY FROM ACOUSTIC DATA

**Fig. 1:**
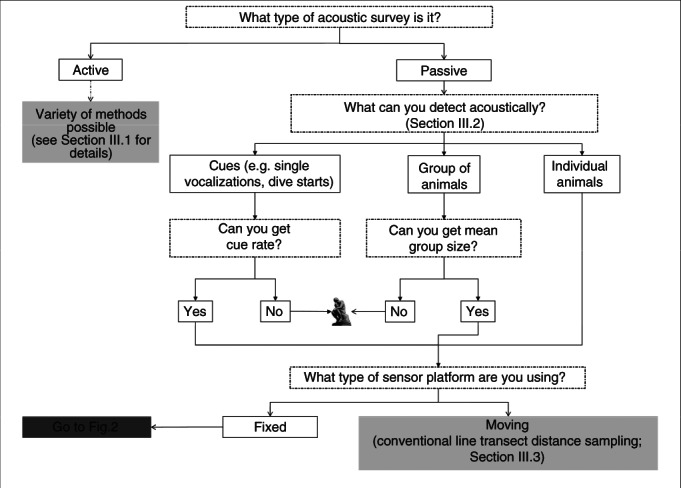
Flowchart representation of the possible approaches to estimate density from acoustic data. The image of Rodin's ‘thinking man’ indicates that further development of solutions is necessary.

**Fig. 2:**
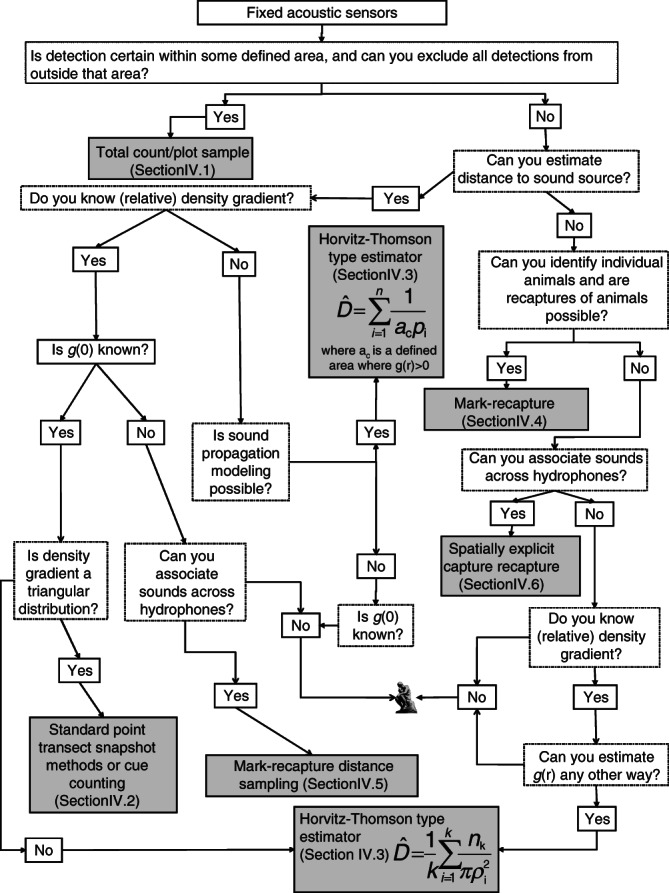
Flowchart representation of the possible approaches to estimate density from fixed passive acoustic sensors. The image of Rodin's ‘thinking man’ indicates that further development of a solution is necessary. *n* represents number of detected animals; *a*_c_ is the area where detection probability is not 0; *p*_i_ is the detection probability of the *i*^th^ animal; *k* is the number of sampling units (e.g. points); *ρ*_k_ is the effective sampling radius of point *k*; g(*r*) is the detection probability at distance *r*.

As should be clear by now, there are many ways that acoustic data could be used to estimate density, depending upon exactly what type of acoustic and auxiliary information is available and what assumptions can be made. In this and the next section we map out the options, including some approaches that seem promising but have not yet been implemented. To help potential users, we present the map as flowcharts (Figs 1 and 2) akin to a taxonomic key, where answering a series of questions leads (hopefully) to a suggested method. However, in some situations, we do not yet know how density could be estimated, and this we represent with an image of Rodin's ‘thinking man’, exhorting us perhaps to think harder about the problem or develop better technological solutions.

### (1) Active acoustic surveys

The first decision point relates to the type of acoustic data collection. Data might be gathered passively, by using a receiver to listen for animal-produced sounds, or actively, by emitting sound and detecting its reflection off animals of interest. Active acoustics has been used widely to estimate animal density, mostly of fish (e.g. Zwolinski *et al*., 2009; Mann *et al*., 2010) and krill (e.g. Cox *et al*., 2011). Generally, active acoustics provides range estimation and localization. However, classification can be extremely difficult, counting individuals might be daunting, and the sound may affect the distribution and movements of the animals. Potential detrimental effects on animals (both target and non-target species) within the sound field are a concern. Risch *et al*. (2012) show an effect on behaviour of humpback whales (*Megaptera novaeangliae*) from fisheries-related sound 200 km away. Surprisingly little is known about these effects in many taxa (e.g. Nowacek *et al*., 2007); however concern about impact, particularly on marine mammals, means that the use of passive acoustics for scientific studies is generally preferred over active in cases where both methods are feasible. Therefore, here we focus on passive acoustics, in which sounds naturally produced by the species of interest are detected and used to make inferences about their populations. Nonetheless we note that the type of information obtained from active acoustics is not intrinsically different from that obtained from passive acoustics (although active acoustics is more like sightings data in the sense that the animals do not need to produce sound to be detected), and hence similar methods could in principle be extended to active acoustic data.

### (2) Type of objects detected acoustically

One fundamental determinant in choosing the analysis method is defining what can be detected acoustically. If one can detect and count individual animals, then animal density can potentially be estimated directly (e.g. Lewis *et al*., 2007). Most common, however, is the application of cue-based methods (i.e. detecting and counting sounds made by the animals), hence requiring cue rates to turn estimates of cue density into animal density (e.g. Marques *et al*., 2009). Alternatively, it is sometimes possible to detect groups of animals, but not be able to determine the number of animals in each group (e.g. Moretti *et al*., 2010). In this case, the density of groups can potentially be determined, but independent information on average group size is required to convert this group density to animal density. If more than one possibility is available, intrinsic variability in cue rates and group sizes might be a determinant in choosing the object of interest. Where only detections of groups or cues are made, but there is no information about cue rates or group sizes, estimation of animal density is difficult; but even if less useful, estimating density of groups, or density of sounds, is still possible.

### (3) Towed acoustic sensors

The fundamental differences between fixed and towed sensors in passive acoustics relates to the geometry of the problem, with fixed sensors akin to point transects, and towed sensors akin to line transects. As for their conventional distance sampling counterparts, this means that animal movement has different influences on the data being collected. While for towed sensors animal movement is usually assumed to be negligible (even if not always true), for fixed sensors this assumption does not hold. Therefore the methods used are necessarily different. Animal movement is also one of the major issues associated with conventional animal surveys, especially when considering methods based on distance sampling. It is usually safe to assume that animals are not counted multiple times from the same transect with towed sensors, simplifying data analysis. However, in fixed surveys one needs to deal explicitly with the fact that one can, and most likely will, detect the same animal multiple times from the same point. Although we briefly review here the use of towed sensors, we subsequently place most of our focus on density estimation using fixed arrays of acoustic devices.

**Fig. 3:**
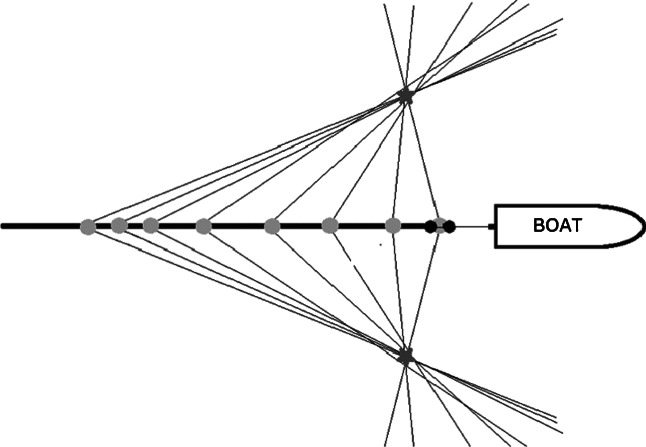
Plan view showing hypothetical bearings to a sound source estimated from time difference of arrival (TDOA) measurements taken at different two-sensor (black dots behind the boat) array positions (grey dots along the track line). Estimated position of the sound-producing animal, assuming it is at the surface or the same depth as the observer, is represented by a grey star. Note that in general left-right ambiguity is not easily resolved, although varying the movement direction of the observation platform helps.

We can estimate density from a moving platform equipped with a recording device using methods akin to commonly used line transects for forest birds. Until recently, and to the best of our knowledge, only sperm whales (*Physeter macrocephalus*) had been the focus of density estimation using towed arrays. In its simplest form a pair of hydrophones cabled to a ship is used to detect vocalizations and obtain a time difference of arrival (TDOA), which in turn is used to estimate a conical bearing angle referenced to the array. Assuming the animals' speed is slow compared to the vessel's speed, successive bearings intersect in space allowing the estimation of a distance (Fig. 3). More sophisticated acoustic arrays, with more hydrophones, can potentially determine distance from a single vocalization (e.g. von Benda-Beckmann *et al*., 2010). The distances are then used to derive conventional line transect estimates of abundance. Leaper, Gillespie & Papastavrou (2000), Hastie *et al*. (2003) and Lewis *et al*. (2007) considered detections to be of individual animals (i.e. assumed animals not to be in clusters). On the other hand, Barlow & Taylor (2005) relied on visual observation for the group sizes. In the above studies, which used towed arrays, the whale depth was not estimable and the calculations of bearing represented a conical angle. Hence the distance obtained is not the distance projected on the sea surface, required for unbiased density estimation using a conventional distance sampling approach, but the three-dimensional slant distance with respect to the ship's track line. Nonetheless, Barlow & Taylor (2005) showed that their sperm whale density estimates are insensitive to the assumed depth because the average distances are quite large and hence the difference in slant and horizontal distance is small compared to the scale of the distances involved. If the potential bias were not negligible, it should be possible to extend standard methods to incorporate an assumed distribution of animal depths. A further alternative is to use a more sophisticated acoustic array or signal-processing algorithm – capable of resolving horizontal and vertical bearing.

SCANS-II used line-transect-based acoustic detections for estimating harbour porpoise (*Phocoena phocoena*) density (SCANS-II, 2008). Recently Gerrodette *et al*. (2011) used towed hydrophones to report conventional distance sampling estimates for vaquita (*Phocoena sinus*). Recent studies have used similar methods for minke whales in Hawaiian waters (T. Norris, personal communication) and for beaked whales in the Azores (J. Gordon, personal communication).

While not having implemented a density estimation exercise, von Benda-Beckmann *et al*. (2010) present a towed system for beaked whale detection. Similarly, Li *et al*. (2009) suggest that their acoustic system could be used to estimate freshwater cetacean densities, namely finless porpoise *Neophocaena phocaenoides*, using similar methods to those which have been used for sperm whales. For this same species, Akamatsu *et al*. (2008) evaluated the probability of detecting an animal from a towed hydrophone array by comparing acoustic and visual observations, putting it in the context of distance sampling surveys. Note that, due to the limited depth of a river, for detections at relatively large distances the slant *versus* horizontal distance measurement error fades in rivers.

Holt (2008) utilized a towed system to identify spawning areas for the red drum *Sciaenops ocellatus*, suggesting that these methods might be developed further to estimate density of some fish species.

## IV. ESTIMATING DENSITY USING FIXED PASSIVE ACOUSTICS: EXAMPLES AND CASE STUDIES

There are a number of possible approaches to estimate density from passive acoustics data collected on fixed sensors. We present those here following the framework presented in Fig. 2.

### (1) Census/strip transects

If all animals within a given area are detected, and animals outside that area can be excluded, then census/strip transect methods are possible. We provide here two application examples, although we note that strictly speaking, these do not represent a census of animals because we need multipliers (e.g. accounting for group size, or proportion of time producing sounds) to produce actual animal density estimates from sound detections.

Moretti *et al*. (2010) present a method based on counting dives of Blainville's beaked whale (*Mesoplodon densirostris*) to estimate their density at the Tongue of the Ocean, Bahamas, using 82 bottom-mounted hydrophones at the US Navy Atlantic Undersea Test and Evaluation Center (AUTEC). Given the sensor array and the species characteristics, dives within the range were assumed to be detected with certainty. Conversely, dives detected outside the range could be excluded. The actual object counted was the onset of echolocation clicking by a group initiating a deep foraging dive – a ‘dive start’. The *n*_d_ dive starts detected during a period of time *T*, coupled with two multipliers, dive rate (*r*_d_), obtained from DTAG data, and average group size *s*, obtained from visual observations, were used to estimate beaked whale density in the area of size *A* by


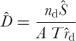
(10)

In a similar exercise in the same area, using animal-based rather than dive-based counting, Ward *et al*. (2012) estimated average density of sperm whales over a 42-day period. Unlike the beaked whale case, sizes of vocalizing groups were obtained directly from the acoustic data, using an algorithm developed by Baggenstoss (2011). Given the sound source level of sperm whales and the spacing of the AUTEC hydrophones, detection of all animals vocalizing within the range was a reasonable assumption. The number of animals detected was combined with an estimate of the proportion of time spent vocalizing by the average whale to obtain an estimate of average sperm whale density during the survey period.

Moretti *et al*. (2010) and Ward *et al*. (2012) differ in the way they dealt with the time dimension. While the former used a cue rate (dive rate), the latter considered inferences regarding groups of animals over, at least conceptually, snapshots of time. These are two different approaches to deal with the fact that recordings occur over time, and hence the same animals could be detected multiple times, as a consequence of undetected animal movement. While in the former case the problem is avoided by using dive starts as the object of interest (and a dive start cannot be recorded twice), in the latter case the snapshot time is chosen such that the same animal is not recorded twice in that period.

A census-related approach is presented by Driscoll (1998), who assessed the extent to which the number of singing male frogs could be taken as an estimate of population size. For the two species studied, *Geocrinia alba* and *G. vitellina*, his method could account for between 76 and 96% of the males in a pond, leading to, at least when coupled with a sex ratio assumption, results close to a census. A similar approach, also requiring accounting for sex ratio and availability was presented by Fischer *et al*. (1997) to estimate grasshopper abundance.

### (2) Distance sampling

Provided distances to detected animals can be obtained, and conventional distance sampling assumptions hold, standard point transect/cue counting methods can be used. McDonald & Fox (1999) used closely related ideas in one of the first attempts to estimate animal density from acoustic data, presenting an estimate of the minimum density of fin whales near the north of Oahu, Hawaii.

Marques *et al*. (2011) presented an example of standard point transect sampling, in particular a cue-counting approach, to estimate right whale *Eubalaena japonica* density in the Bering Sea. Given *n*_u_ detected right whale calls in *T* hours, animal density was estimated by


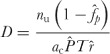
(11)

where *a*_c_ is the size of the covered area, 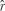 the estimated call rate (in calls per hour), 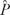 the estimated detection probability of a call produced within area *a*_c_, and 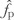 the estimated proportion of false positives (assumed to be zero in their example). Note that 
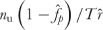
 corresponds to *n* in equation (2), i.e. the number of detected calls must be ‘corrected’ to represent the number of detected animals.

This novel approach used particular characteristics of sound propagation in shallow waters of the Bering Sea continental shelf, which allowed distances to detected calls to be obtained using the data collected at the individual sensors, therefore avoiding the need to associate detections across multiple hydrophones to obtain distances. (‘Association’ refers to the process of determining which sounds received on multiple sensors, or at multiple times due to multipath propagation, arose from the same source sound instance.) To convert call density to animal density an independent call rate estimate was derived from acoustic recordings made in the vicinity of groups of known size. Here measurement error due to unknown depth is unlikely to have been responsible for bias, because depth variation is small (∼200 m) compared to the distances measured (20–80 km).

While useful as a proof-of-concept, this example is a sub-optimal demonstration of density estimation in two respects. First, the cue rate used was obtained for a different time and place than the survey, and the sample size available was relatively small. Instead, we recommend that the cue rate be estimated concurrently with the survey data, over a large and random sample of animals. In doing so one ensures that the cue rate estimate is representative for the survey period, and variations of cue rate with respect to unmeasured covariates can be safely ignored. Second, only three sensor locations were used, clearly not enough to ensure that the required assumption of a known distribution of animals with respect to the sensors holds, or to allow reliable extrapolation from density in the vicinity of the sensors to density over a larger area of interest. Further, because the variance in *n*_u_ was obtained using the empirical variance estimate in counts over different sensors, it is a very unreliable estimate of the variance, and hence extreme caution should be used in the interpretation of reported precision measures. A much more reliable approach would require an array of independent sensors capable of determining range to detected objects, located through an area of interest according to a (systematic) random survey design. An example is a set of 24 ocean bottom seismometer (OBS) arrays, which were used to localize fin whales *Balaenoptera physalus* to the south west of the Iberian Peninsula (Harris, 2012). OBS arrays were also used to track fin whales (Rebull *et al*., 2006) and blue whales *Balaenoptera musculus* (Dunn & Hernandez, 2009) and represent a potential tool for density estimation of some species.

### (3) Detection function estimated from auxiliary data

The previous examples only work in the particular setting where distances can be obtained from single sensors or clusters of closely spaced sensors, each operating as a single unit. In most cases, that is not easily done. If instead one can obtain an estimate of the detection function from some independent data set or from a model, density can still be estimated from a widely spaced set of independent sensors (a ‘sparse array’) using distance-sampling-related approaches. Explicit assumptions about the distribution of animals with respect to the sensors are still required, and the estimators used will still be of the same form as equation (2). It is just the method used to estimate the detection function, and hence *p*, that differs. Lacking a better name, we refer to these as ‘Horvitz-Thompson (HT) type’ estimators [e.g. Borchers & Burnham (2004, p. 10), note that, strictly, all forms of estimators derived from equation (2) might be seen as such] in the sense that the probability of including a sampling unit in the sample is estimated rather than known as in conventional HT estimators (Horvitz & Thompson, 1952).

#### (a) Trials using sounds for which location is known

Marques *et al*. (2009) presented an example in which data from acoustic recording tags (DTAGs) attached to free-ranging Blainville's beaked whales at AUTEC were integrated with data from 82 bottom-mounted hydrophones to estimate the detection function of the bottom-mounted hydrophones for detecting echolocation clicks. For each click produced, the DTAG provided click emission time, animal depth, and relative animal orientation in space with respect to the surrounding bottom-mounted hydrophones. The detection or non-detection of each click emitted by the tagged whale in the surrounding bottom-mounted hydrophones was assessed. This allowed the detection function to be estimated in a generalized additive modelling framework. From this, the average probability of detecting a click was estimated using a Monte Carlo procedure. The estimated probability of detection was then used to derive a density estimate 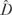 from a separate 6 days of monitoring data. Additional information required was (*i*) automated counts of clicks at the bottom-mounted hydrophones (*n*), (*ii*) an estimate of the false positive rate (*f*), obtained by manual inspection of a systematic random sample of monitoring data, and (*iii*) a cue rate (*r*) obtained from the tag data. The density estimator used was


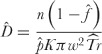
(12)

which is essentially the same as equation (3) above, where *Kπw^2^* is the surveyed area *a*, and *T* is the recording time. Note *K* was 82 and *w* = 8 km, a distance beyond which it was safely assumed no beaked whale click would be detected.

Another example of known animal locations using visual observers is provided by Kyhn *et al*. (2012). An acoustic tracking array might also potentially be used to yield known animal locations.

Setting up artificial trials might represent an alternative to using vocalizations produced by free-ranging animals *in situ*, either by: (*i*) using synthetic sounds or (*ii*) placing acoustic sensors at a range of known distances from captive animals. These options have different shortcomings. Regarding synthetic sounds, adequate sounds must be generated, either by synthesizing the sounds or by playing back appropriate animal recordings. Multiple factors need to be considered, including source level, sound emission beam pattern, depth distribution, orientation distribution, etc. This is a potential research topic. Regarding animals placed at known locations, the vocal characteristics of a captive animal might be different from animals in the wild, and in any case, not many species are kept captive.

Thompson *et al*. (2009) presented a methodology for density estimation suitable for vocal mammals in forested habitats, also based on distance sampling concepts. The technique was illustrated using African forest elephant (*Loxodonta africana cyclotis*) sounds in the Central African Republic, and applied to another elephant population in Ghana (Thompson, Schwager & Payne, 2010).

#### (b) Acoustic modelling

Rather than using measurements, one can derive the detection function based on first principles from the physics involved coupled with knowledge, or at least reasonable assumptions, about the acoustic system at hand, namely the detection and classification system involved, the ambient characteristics and the species vocal behaviour. Kimura *et al*. (2010) and Küsel *et al*. (2011) provide two examples of such an approach. The detection function was obtained using a sound propagation model based on the sonar equation following the approach of Zimmer *et al*. (2008). Both of these studies are interesting, but due to their clear proof-of-concept status, presented potential problems which are worth considering in detail, as they are general enough to be applicable to other situations.

Both Kimura *et al*. (2010) and Küsel *et al*. (2011) considered single sensors, and the consequences of doing so need to be explored. While clearly stated as a handicap by Küsel *et al*. (2011), use of a single sensor has potential problems ignored by Kimura *et al*. (2010). If a single sensor is used, it is much less likely that the implicit assumption regarding the distribution of animals (a triangular distribution for distances to available cues) holds. In particular for Kimura *et al*. (2010), where sensors (multiple sensors, but analysed as independent single sensors) were placed in the middle of a river, it is expected that animals might not distribute themselves independently of the sensors' locations. This will result in unpredictably biased estimates of detection probability, and hence density. Note that both studies considered a simulation approach to obtain the estimated detection probability. Therefore a known distribution of animals with respect to the sensor is crucial. Only by defining this distribution can one simulate the animal's location, a fundamental step to estimating the detection probability. Further, variance estimation is not straightforward if only a single sensor is used, as there is no replication in the number of detected cues. Typically this has been dealt with by using a distributional assumption for the number of detected cues (e.g. Küsel *et al*., 2011), but as before, a much more robust approach would be to estimate variance in counts across sensors.

An additional shortcoming shared by these studies is the small data set used to characterize the detector performance, representing a tiny fraction of the entire survey period and in particular not a random sample over time. If the focus is on the actual density estimates rather than the illustration of an approach, a considerably larger data set should be used to characterize detector performance.

To estimate the detection function, we prefer methods based on empirical measurements (e.g. Marques *et al*., 2009), rather than the model-based methods (e.g. Kimura *et al*., 2010; Küsel *et al*., 2011). This preference is based on our concern that sometimes the assumptions used to implement the models are not fulfilled or have unforeseen consequences, while empirical measurements, though not ideal to explain the process generating the data at hand, are less likely to be affected by otherwise unexpected peculiarities of the system under study.

### (4) Mark-recapture

In some cases, individual identification is possible from the detected sounds, e.g. frogs *Rana clamitans* (Bee *et al*., 2001), manatees *Trichechus inunguis* (Sousa-Lima, Paglia & Fonseca, 2002), bearded seal *Erignathus barbatus* (Van Parijs & Clark, 2006), toadfish *Halobatrachus didactylus* (Amorim & Vasconcelos, 2008), birds (Cheng, Sun & Ji, 2010); see Fox (2008) for a review on individual recognition from acoustic signals. An estimate of abundance then is possible using a conventional mark-recapture approach. Adi, Johnson & Osiejuk (2010) present an example application, using state of the art detection and classification algorithms based on hidden Markov models, coupled with simple mark-recapture models.

### (5) Mark-recapture distance sampling

Widely applied in visual surveys of cetaceans (e.g. Laake & Borchers, 2004), to the best of our knowledge mark-recapture distance sampling approaches have not been implemented using acoustic data. One of the reasons might be that these approaches address primarily perception bias (observer fails to detect animal), but acoustic data suffer mostly from availability bias (i.e. animal is silent). It is conceivable that, although an option, MRDS will not receive much attention in exclusively acoustic surveys. We note however that MRDS seems a promising approach to estimate availability bias in the context of combined acoustic and visual surveys, an idea that dates back to Fristrup & Clark (1997). The low use of MRDS is also expected due to the fast development of closely related spatially explicit capture-recapture (SECR) methods, which we address next (in fact, conceptually MRDS represents a special case of SECR, with known locations, as described by Borchers, 2012).

### (6) Spatially explicit capture-recapture

If distances to detected animals cannot be directly obtainable from the data, but the same sound can be detected and associated across multiple sound sensors, SECR methods (e.g. Borchers & Efford, 2008) are a natural choice. Synchronization of the multiple sensors used facilitates sound association, but the methods will work provided sounds can be matched across sensors irrespective of the sensors being precisely time aligned.

These methods have been recently applied to both birds (Dawson & Efford, 2009) and whales (Marques *et al*., 2012; Martin *et al*., in press), considering animal- and cue-based approaches, respectively. In both cases it was assumed that detection of a sound produced directly above a sensor was certain. Note however that SECR methods deal well with the situation in which *g*(0) is less than 1, as the data contain information that allows its estimation. An implementation of these methods to estimate the density of a South African frog (*Arthroleptella lightfooti*) is also underway (D. L. Borchers, personal communication).

Dawson & Efford (2009) estimated ovenbird *Seiurus aurocapilla* density using acoustic data collected on a four-sensor array. The detection model explicitly considered the incorporation of received signal strength as a proxy for distance. The inclusion of this information enabled efficiency gains over conventional capture histories.

Marques *et al*. (2012) used SECR to estimate the density of minke whales (*Balaenoptera acutorostrata*) near Kauai, Hawaii. The data consisted of minke ‘boing’ sounds detected over 16 hydrophones at the US Navy's Pacific Missile Range Facility (PMRF). Boings from six different time periods, of 10 min each, were combined to obtain an average density estimate over the full hour period. Sounds were manually associated across hydrophones, leading to vectors of capture histories, which were then used within the SECR framework simultaneously to estimate the ‘boing’ detection function and density. Strictly speaking, because there is no reasonable estimate of ‘boing’ production rate available, the obtained estimate was for ‘boing’ density (over a period of time) rather than for actual minke whale density. But once a cue rate is available, dividing the cue density by cue rate will provide an estimate of minke whale density. This will only be valid provided one can safely assume that the cue rate was valid during the survey period, which might be the object of its own dedicated study. Martin *et al*. (in press) expands on this preliminary study and estimates minke whale abundance at PMRF based on a larger data set.

## V. DISCUSSION

Passive acoustics is increasingly used for estimating animal density, with a number of applications published in the last few years. All of the density estimation methods presented here have explicit, and often implicit, assumptions. When applying the methods, investigators should assess whether these assumptions hold to a reasonable extent, and discuss the consequences of their possible failure. Density estimates based exclusively on acoustic data have already been presented for some taxonomic groups including insects (e.g. Fischer *et al*., 1997), cetaceans (e.g. Marques *et al*., 2009), birds (e.g. Dawson & Efford, 2009; Adi *et al*., 2010) and elephants (e.g. Thompson *et al*., 2009). For other groups, like freshwater (e.g. Anderson, Rountree & Juanes, 2008) and marine fish, absolute density estimation has not yet been attempted, but will likely happen in the near future (see the extensive review regarding passive acoustics in fisheries provided by Gannon (2008); also Luczkovich *et al*. (2008) and Mann *et al*. (2010)). Gardiner, Hill & Chesmore (2005) suggested acoustics-based distance sampling methods might be a plausible way to estimate grasshopper density [note that Fischer *et al*. (1997) had already presented such an example]. Some studies (e.g. Royle & Link, 2005) have also used anuran call index data to illustrate estimators of relative abundance in a model-based approach, but given that these do not consider an absolute measure of abundance we have not given them emphasis here.

A common application for density estimation is to determine whether a given population has increased or decreased significantly. Often density estimates between two time periods might be statistically significantly different, but the biological significance of that difference is hard to assess. This difficulty stems from the fact that the size of wild populations typically oscillates over time, even in the absence of major environmental changes. The (most often unknown) intrinsic variance of that oscillation pattern might lead one to interpret observed differences in estimated density as a true effect, if the time scale is not properly considered. Acoustics surveys, by allowing the collection of data over long time periods, might be particularly well suited to evaluating these natural oscillations.

Projects like Listening to the Deep Ocean Environment (LIDO), currently the only live worldwide PAM system available (André *et al*., 2011), anticipate the use of acoustic data in real time at large spatial and temporal scales. This was hard to imagine only a few years ago, and is bound to have impacts on how acoustic-based research is conducted. Some large acoustic-based density estimation projects are underway, including the SAMBAH project (http://www.sambah.org/), which involves the deployment of over 300 static porpoise detectors (C-PODs) at a systematic random grid of locations through the Baltic Sea to estimate harbour porpoise density. Gedamke & Robinson (2010), although not attempting density estimates, provided an example of a field of sensors from which robust density estimates could be obtained. We envisage similar sets of ‘cheap’ fixed (or floating) sensors systematically spaced over regions of interest, ideally with ranging capabilities, to be ideal settings for density estimation.

We hope that this review provides a general understanding of the kinds of data required to derive density estimates from acoustics-based methods, as well as the components that are the principal contributors to the overall variance estimates. This will allow further development and application of these techniques, which we anticipate will be used increasingly often in the future.

### (1) Calibration studies and other approaches

We presented a framework for estimating animal abundance from passive acoustics, but some approaches do not fit cleanly into that framework. We list some of those here.

Based on acoustic data, and a number of assumptions, McCauley & Jenner (2010) estimated the number of pygmy blue whales (*Balaenoptera musculus brevicauda*) migrating through an area in Western Australia. In situations where the actual number of animals is known, or at least there is independent information regarding density, and there is a potential sound signal to be recorded, a calibration approach might be considered to predict density when the direct information on density is not available but the sound signal can be recorded. A regression framework could then be used to predict density as a function of an acoustically derived abundance index. Calibrations have also been used to predict abundance from calling indices in anurans (Shirose *et al*., 1997). Grafe & Meuche (2005) suggest that when better methods are not possible, this might be the best option for anuran surveys. A similar idea was used by Van Parijs *et al*. (2002) to predict dolphin group size. This was also the concept behind a proposed approach in which sound in the frequency band produced by fin whales could be calibrated to whale density, using a simulation approach (Mellinger *et al*., 2009). Hagstrum, Webb & Vick (1988) have estimated insect density in stored cereal grains, by calibrating received sounds from containers with unknown insect density using sounds recorded from containers of known insect density. Hugel (2012) presents an additional calibration example with insects. We note that density may not be linearly related to sound production, and hence for reliable calibration, sound levels need to be measured over a range of densities, preferably encompassing those of interest. Also, if the relationship plateaus at either high or low density, then reliable density estimation at the corresponding sound levels will be impossible.

An alternative approach suggested by Whitehead (2009) considers the use of points along a line. At each point presence-absence of sounds from the species of interest is recorded. Coupled with some strong assumptions regarding animal distribution and the shape of the acoustic detection function, this setting allows density estimation. While the approach is attractive as the field methods are simple, it is not easy to generalize, nor useful at high densities in which most sensors detect the presence of the species under study. Further, it is sensitive to departures from the animal distribution assumption. The methods are developed further by Horrocks, Hamilton & Whitehead (2011). Both references provide simulation-based detailed considerations about when the approach could be useful.

Another less-than-ideal situation where density estimation may be possible is where there is a dispersed field of sensors, each capable of determining the bearing to detected sounds, but not the range. One potential example is the U.S. Navy SOSUS arrays, which are composed of sets of beamforming (i.e. directional) sensor arrays that were designed for tracking submarines. These arrays can also be used to detect cetaceans that produce low-frequency sounds (Clark, 1995; Stafford *et al*., 2009), and due to secrecy issues it may be that only direction and count data are available. Given some strong assumptions about the detection process [radial symmetry, *g*(0) known, some overlap of detections between sensors], and the spatial distribution of animals (that it varies smoothly over space), it is theoretically possible to simultaneously estimate the density surface and detection function. Estimation of the detection function component may be rendered more reliable by incorporating elements of acoustic modelling discussed earlier (Section IV.3b). Also, with calibrated sensors, received sound level may provide a useable proxy for distance, enabling distance sampling approaches to be used (Section IV.2).

We have framed methods in terms of towed *versus* fixed sensors. An intermediate scenario is the use of slow moving platforms, like gliders or drifting sensors. Free-floating devices for which a time series of locations are available, such as a global positioning system (GPS)-enabled sonobuoy (Hayes *et al*., 2000), fall somewhere between fixed and towed sensors. While over large time scales these are moving sensors, at small time scales they might be considered fixed. Such devices allow data collection without the need for mooring or towing sensors, at the expense of having to handle the slow drift-related movement of sensors and the (potentially relatively fast) animal movement. Ocean gliders also fall into this category – although their movement is directed, it is very slow, less than the travel speed of many marine mammals. On the US West coast, experiments are being conducted with free-drifting buoy recorders with a vertical hydrophone array for point-transect sampling to estimate cetacean abundance (J. Barlow, personal communication).

### (2) Accuracy of density estimates

In general, a reasonably large sample size is desired to estimate the random components required for the density estimator. Some species allow more accurate density estimates than others. Factors that influence the accuracy of acoustic density estimates depend on the estimation method chosen, but generally include small variance in the parameters that make up the density estimate. These factors can include the following. (*i*) Small variance in the cue rate (rate of sound production), e.g. foraging sounds, which are often made quite regularly at certain times of day. (*ii*) Small variance in the sound level emitted by animals; again, song during the breeding season and foraging sounds are typically produced at relatively high, consistent sound levels. (*iii*) Accurate detection, including low false-positive and false-negative rates for a given signal-to-noise ratio, normally leads to low variance in detectability (i.e. in *p* and *f*). For fixed sensors, the biggest component of variance might be between sensor variability. This highlights the importance of traditional survey design issues such as transect placement and stratification.

### (3) Getting multipliers right

As illustrated by the examples, density estimators often need multipliers which are obtained from data ancillary to the main survey. Examples might include a cue rate or the proportion of false positives. If at all possible, these multipliers should be obtained concurrently with the survey data, as this considerably simplifies inferences. Otherwise, one needs to ensure that either (*i*) the multiplier was collected under the same conditions that were observed during the survey or (*ii*) data are collected to model the relation between the multiplier and any relevant covariates, to predict the multiplier value under the actual observed survey conditions. Marques *et al*. (2009) assumed that the estimated detection function was valid for the survey data, even though the survey was collected over a 6-day data set and the data used for the estimation of the detection function came from DTAGs, and hence presumably periods of good weather conditions (animals are only tagged under good weather). If the data set used to estimate the detection function was collected under good weather and low-noise conditions, a higher probability of detection would be anticipated as compared to a period of elevated ambient noise due to such conditions as high wind and/or rain. Under this scenario, bias in density estimates would follow: the true detection function operating during the survey would be overestimated, and the corresponding density underestimated. If the data sets are not collected at the same time or under the same conditions, a model relating the relevant parameter with appropriate covariates might be an option. As an example, one might model detection function as a function of time of day, and use the time of day from the actual survey rather than the time of day from when the detection function was estimated. Naturally, this brings an additional layer of complexity, and extra uncertainty, to the estimation process. Ward *et al*. (2011) provided an evaluation of the influence of ambient noise in the detector performance under the settings described by Marques *et al*. (2009), noting that, under the scenario considered, in particular regarding the very deep hydrophones used, the bias appears to be small. Nonetheless, for shallower sensors, wind speed has been shown to influence ambient noise (e.g. Baumgartner & Fratantoni, 2008; Marques *et al*., 2011), and may have to be accounted for.

Cue rate (number of cues per unit time) is a special multiplier required for any cue-based method of density estimation. Cues might be as diverse as dive starts, clicks, calls, songs, or any other event produced by the animals that might be detected acoustically. Naturally, cue rates varying over time or space will have impact in comparisons across time or space (or both). This variation might be due to say behavioural state or sex-ratio differences over space and time, and must be accounted for. Cue rates are not well known for most species, and we anticipate future research focused on estimating and modelling these as a function of additional covariates such as time of day, season, sex, behaviour, etc. The cue rate also depends on variables that are usually not observed during the survey period. As an example, animals might produce cues at very different rates depending on behavioural state, hence biasing results if cue rates are estimated under a behavioural state other than that observed during the survey itself. This adds complexity, as it necessitates prediction of the cue rate for the animals in the study area while the survey was underway. Tags deployed on animals, like the A-Tag (Akamatsu *et al*., 2005), the Acousonde (Burgess, 2009), and the DTAG (Johnson & Tyack, 2003), can be extremely valuable for estimation of cue rates. Further developments in this area are essential, given that reliable and precise estimates of cue rate are fundamental to obtaining density estimates (see Marques *et al*., 2011, for an example). If animals produce different types of sounds, or if cue rate varies over time, there are clear benefits in focusing inference on cues or time periods for which the variability across animals is low. Note however that as we are interested in a mean cue rate, provided the sample size is large enough we can always obtain it with high precision. On the other hand, if the survey period itself is short, then whether the long-term average is reasonable or not becomes an issue. Therefore, given that the variability in cue rate is directly reflected in the variance of the density estimate, careful consideration of what and when to survey might lead to significant increases in efficiency. For example, while Blainville's beaked whales show a behaviour that suggests stable echolocation click rates (e.g. Baird *et al*., 2008), a currently unidentified beaked whale seems to click only at night (McDonald *et al*. 2009). Large variability in acoustic behaviour, and complex relations with biotic and abiotic variables, has been described for pinniped species (Van Opzeeland *et al*., 2010). Animal-produced sounds could depend on a large number of factors, as diverse as water temperature (e.g. Amorim *et al*., 2006) or time of day (Bridges & Dorcas, 2000). In the worst-case scenario, cue rate might even depend on density (e.g. Penteriani, Gallardo & Cazassus, 2002; Amorim *et al*., 2011). Such a relationship would often be expected for sounds used for social communication. This means one is faced with a circular problem: needing cue rate to estimate density, and needing density to estimate cue rate. In such a case, there is no alternative to obtaining the cue rate during the survey.

Another particular consideration that relates to cue rates is that the cue rate should account for silent periods. As an example, the time period used to estimate the cue rate should be long enough such that the obtained cue rate is an adequate average over the survey period. One cannot find animals acoustically and then follow them for a short period to record cue rate, as animals vocalizing in the first place are most likely more acoustically active than animals not detected. A closely related point is that potentially only a fraction of the population might be detectable using acoustics (e.g. singing males in some bird, frog or whale species), and hence care is needed in the interpretation of the results in terms of overall population estimates. If cue rates already account for this fraction of the unavailable population (say by selecting a random sample of all animals for estimating cue rate, including potentially silent animals), then this issue is automatically dealt with in the estimation process *via* the cue rate.

### (4) Future research areas

There are a number of topics that we anticipate will become fruitful research areas in this rapidly developing field.

Automatic detection and statistical classification of sounds will continue to be fertile topics for further research, as these will allow the efficient processing of large quantities of data (e.g. Parsons & Jones, 2000; Acevedo *et al*., 2009), making methods cheaper and faster, and hence more appealing. Passive acoustic detection and classification requires knowledge of a species' vocal behaviour, yet for many species these data do not exist. To close this knowledge gap, extensive research into the acoustic behaviour of species of interest is necessary.

Custom-designed acoustic density estimation hardware is another area of potential development. In particular, a reliable, portable, inexpensive autonomous sensor or array of sensors capable of ranging would be most desirable. This would allow reasonable survey designs for abundance estimation to be implemented and the data analysed using conventional distance sampling methods. An array of closely spaced sensors, effectively operating as a single sampling point, which processes sound and allows distances to sounds of interest to be obtained is the ideal tool. Mennill *et al*. (2006) present a possible precursor of such a system.

There are several examples of automated acoustic monitoring of birds (e.g. Hobson *et al*., 2002; Acevedo & Villanueva-Rivera, 2006; Celis-Murillo, Deppe & Allen, 2009; Johnson *et al*., 2009). We anticipate similar developments suited and optimized for other taxa or environments, and in particular with density estimates in mind.

It is likely that many more species than currently known produce sounds, lending themselves to acoustic methods. Some of these sound-producing species have been found almost by accident (e.g. Lowe & Skelton, 2008), and hence dedicated exploratory monitoring might be useful for some taxa and areas, opening the door to the survey methods reviewed here.

Survey design for acoustic methods is a poorly explored area. In particular, sensor spacing and relative location, to optimize data collection for SECR, will likely become an important topic for future research. Something worth considering *a priori* is whether the scale at which sound is transmitted and hence can be detected, coupled with realistic scenarios for sensor deployment, provides the relevant information to obtain density estimates at the desired spatial scale. Contrasting the scale at which acoustic or visual data might be collected might also help in choosing between different methods. Surveys for which a field of cheap sensors is used, and some sensors are moved around (increasing spatial coverage) but some remain fixed (allowing better power for detecting trends) seems a good compromise.

Given that good spatial coverage is often hard to achieve with fixed sensors, we also anticipate the use of gliders (e.g. Moore *et al*., 2007) to become widespread, providing reasonable spatial coverage along a designed set of transects at moderate expense (certainly much cheaper than towing a hydrophone behind a ship). Analysis methods might need to be developed for such special slow-moving platforms, which are neither stationary nor move fast enough to eliminate the risk of recounting the same animal.

One noticeable feature of the work reviewed here is the absence of published references in which truth, in terms of density and abundance, is known. While such situations are difficult to identify, it would be of great benefit to have case studies that provide a means of verification. Much has been learned from verifiable case studies of visually based surveys, providing insight into the generality of their application, the validity of their assumptions, and the consequences of their failure, under different settings (e.g. Anderson *et al*., 2001; sections 8.2. and 8.3 in Buckland *et al*., 2001; Conn *et al*., 2006).

## VI. CONCLUSIONS

1. Passive acoustic detection and localization of animal calls is an expanding field, and will likely become increasingly used for taxonomic groups in which species naturally produce sound, including mammals, birds, fishes, amphibians, and insects.
2. Using passive acoustic data for density estimation seems a natural and efficient alternative to visual methods for many taxa and habitats, and will likely see increased use and fast development in the coming years. This is particularly true underwater, where sound travels better than in air, while visual methods are less effective.
3. Linking acoustic production to species' behaviour is fundamental. Some recorded sounds have yet to be assigned to a particular species, and the sounds of many species have yet to be discovered and described. Further, information regarding species-specific sound source levels and directionality is often helpful. In particular, estimating cue rates and relating cue rate to environmental covariates is fundamental for cue-based methods.
4. Analysis methods based on distance sampling and spatially explicit mark-recapture methods are promising candidates for the analysis of acoustic data for density estimation; alternatively, the information to estimate the detection function may come from auxiliary information or analysis rather than measured distances to detected objects of interest.
5. Methods based on modelling sound propagation are useful, but they need to be checked against empirical data to avoid biased results. On the other hand, these model-based methods might be better suited than empirical methods to explain the underlying process generating the patterns observed in the data.
6. Required multipliers such as cue rates should be obtained under actual survey conditions. Otherwise, survey results are always subject to the potential issue that the multiplier depends on unmeasured covariates that may differ between the time and place where the multiplier and survey data were collected, leading to bias.
7. Improved detection and classification algorithms are desirable and are a fruitful research area at the interface of acoustics, signal processing, and statistics. Nonetheless, given accurate characterization of a system's performance, density estimation is possible even using sub-optimal systems.
8. Demand exists for hardware tailored for density estimation. In particular, a device with on-board sound processing and capable of estimating range to detected objects would allow conventional analysis methods to be used in a straightforward way. Tags optimized for cue-rate estimation would also be most welcome.
9. Acoustically based surveys are ideally suited for drawing inferences over time, given that data can be collected (continuously or not) over long time periods. Therefore, acoustic-based methods might be ideal to address trends over time at multiple scales.
10. Survey design is key. In particular, choosing sensor placements that adequately sample the variance present in a population is important, as is choosing time periods in which the variances of cue rate and sound level are relatively small.
11. Application of the methods under known truth scenarios is welcome, as it will allow confirmation of the usefulness of the methods under real settings.

## References

[b1] Abbot TA, Premus VE, Abbot PA (2010). A real-time method for autonomous passive acoustic detection-classification of humpback whales. The Journal of the Acoustical Society of America.

[b2] Acevedo MA, Corrada-Bravo CJ, Corrada-Bravo H, Villanueva-Rivera LJ, Aide TM (2009). Automated classification of bird and amphibian calls using machine learning: a comparison of methods. Ecological Informatics.

[b3] Acevedo MA, Villanueva-Rivera LJ (2006). Using automated digital recording systems as effective tools for the monitoring of birds and amphibians. Wildlife Society Bulletin.

[b4] Adi K, Johnson MT, Osiejuk TS (2010). Acoustic censusing using automatic vocalization classification and identity recognition. The Journal of the Acoustical Society of America.

[b5] Akamatsu T, Matsuda A, Suzuki S, Wang D, Wang K, Suzuki M, Muramoto H, Sugiyama N, Oota K (2005). New stereo acoustic data logger for free-ranging dolphins and porpoises. Marine Technology Society Journal.

[b6] Akamatsu T, Wang D, Wang K, Li S, Dong S, Zhao X, Barlow J, Stewart BS, Richlen M (2008). Estimation of the detection probability for Yangtze finless porpoises (*Neophocaena phocaenoides asiaeorientalis*) with a passive acoustic method. The Journal of the Acoustical Society of America.

[b7] Alldredge MW, Simons TR, Pollock KH (2007). A field evaluation of distance measurement error in auditory avian point count surveys. Journal of Wildlife Management.

[b8] Amorim M, Simões J, Almada V, Fonseca P (2011). Stereotypy and variation of the mating call in the Lusitanian toadfish, *Halobatrachus didactylus*. Behavioral Ecology and Sociobiology.

[b9] Amorim M, Vasconcelos RO (2008). Variability in the mating calls of the Lusitanian toadfish *Halobatrachus didactylus*: potential cues for individual recognition. Journal of Fish Biology.

[b10] Amorim MCP, Vasconcelos RO, Marques JF, Almada F (2006). Seasonal variation of sound production in the Lusitanian toadfish, *Halobatrachus didactylus*. Journal of Fish Biology.

[b11] Anderson DR, Burnham KP, Lubow BC, Thomas L, Corn PS, Medica PA, Marlow RW (2001). Field trials of line transect methods applied to estimation of desert tortoise abundance. Journal of Wildlife Management.

[b12] Anderson KA, Rountree RA, Juanes F (2008). Soniferous fishes in the Hudson river. Transactions of the American Fisheries Society.

[b13] André M, van der Schaar M, Zaugg S, Houégnigan L, Sánchez A, Castell J (2011). Listening to the deep: live monitoring of ocean noise and cetacean acoustic signals. Marine Pollution Bulletin.

[b14] Aubauer R, Lammers MO, Au WWL (2000). One-hydrophone method of estimating distance and depth of phonating dolphins in shallow water. The Journal of the Acoustical Society of America.

[b15] Baird RW, Webster DL, Schorr GS, Mcsweeney DJ, Barlow J (2008). Diel variation in beaked whale diving behaviour. Marine Mammal Science.

[b16] Baggenstoss PM (2011). An algorithm for the localization of multiple interfering sperm whales using multi-sensor time difference of arrival. The Journal of the Acoustical Society of America.

[b17] Bardeli R, Wolff D, Kurth F, Koch M, Tauchert K-H, Frommolt K-H (2010). Detecting bird sounds in a complex acoustic environment and application to bioacoustic monitoring. Pattern Recognition Letters.

[b18] Barlow J, Taylor B (2005). Estimates of sperm whale abundance in the northeastern temperate Pacific from a combined acoustic and visual survey. Marine Mammal Science.

[b19] Barnes C, Best M, Johnson F, Phibbs P, Pirenne B (2008). Transforming the ocean sciences through cabled observatories. Marine Technology Reporter.

[b20] Baumgartner MF, Fratantoni DM (2008). Diel periodicity in both sei whale vocalization rates and the vertical migration of their copepod prey observed from ocean gliders. Limnology and Oceanography.

[b21] Baumgartner MF, Van Parijs SM, Wenzel FW, Tremblay CJ, Esch HC, Warde AM (2008). Low frequency vocalizations attributed to sei whales (*Balaenoptera borealis*. The Journal of the Acoustical Society of America.

[b22] Bee M, Kozich C, Blackwell K, Gerhardt HC (2001). Individual variation in advertisement calls of territorial male green frogs, *Rana clamitans*: implications for individual discrimination. Ethology.

[b23] von Benda-Beckmann A, Lam F, Moretti D, Fulkerson K, Ainslie M, van IJsselmuide S, Theriault J, Beerens S (2010). Detection of Blainville's beaked whales with towed arrays. Applied Acoustics.

[b24] Bingham G (2011). Status and Applications of Acoustic Mitigation and Monitoring Systems for Marine Mammals.

[b25] Blumstein DT, Mennill DJ, Clemins P, Girod L, Yao K, Patricelli G, Deppe JL, Krakauer AH, Clark C, Cortopassi KA, Hanser SF, McCowan B, Ali AM, Kirschel ANG (2011). Acoustic monitoring in terrestrial environments using microphone arrays: applications, technological considerations and prospectus. Journal of Applied Ecology.

[b26] Borchers DL (2012). A non-technical overview of spatially explicit capture-recapture models. Journal of Ornithology.

[b27] Borchers DL, Buckland ST, Zucchini W (2002). Estimating Animal Abundance.

[b28] Borchers DL, Burnham KP, Buckland ST, Anderson DR, Burnham KP, Laake JL, Borchers DL, Thomas L (2004). General formulation for distance sampling. Advanced Distance Sampling.

[b29] Borchers DL, Efford M (2008). Spatially explicit maximum likelihood methods for capture-recapture studies. Biometrics.

[b30] Borchers DL, Marques TA, Gunnlaugsson T, Jupp PE (2010). Estimating distance sampling detection functions when distances are measured with errors. Journal of Agricultural, Biological, and Environmental Statistics.

[b31] Bower JL, Clark CW (2005). A field test of the accuracy of a passive acoustic location system. Bioacoustics.

[b32] Brandes TS, Naskrecki P, Figueroa HK (2006). Using image processing to detect and classify narrow-band cricket and frog calls. The Journal of the Acoustical Society of America.

[b33] Bridges AS, Dorcas ME (2000). Temporal variation in anuran calling behavior: implications for surveys and monitoring programs. Copeia.

[b34] Bucci M, Petryszyn Y, Krausman PR (2010). Occurrence and activity of bats at three national monuments in central Arizona. The Southwestern Naturalist.

[b35] Buckland ST (2006). Point transect surveys for songbirds: robust methodologies. The Auk.

[b36] Buckland ST, Anderson DR, Burnham KP, Laake JL, Borchers DL, Thomas L (2001). Introduction to Distance Sampling – Estimating Abundance of Biological Populations.

[b37] Buckland ST, Anderson DR, Burnham KP, Laake JL, Borchers D, Thomas L (2004). Advanced Distance Sampling.

[b38] Burgess WC (2009). The Acousonde: a miniature autonomous wideband recorder. The Journal of the Acoustical Society of America.

[b39] Burgess WC, Tyack PL, LeBoeuf BJ, Costa DP (1998). A programmable acoustic recording tag and first results from free-ranging northern elephant seals. Deep Sea Research II.

[b40] Cato DH (1998). Simple methods of estimating source levels and locations of marine animal sounds. The Journal of the Acoustical Society of America.

[b41] Cato D, McCauley R, Rogers T, Noad M, McMinn T (2006). Passive acoustics for monitoring marine mammals – progress and challenges. Proceedings of ACOUSTICS 2006.

[b42] Celis-Murillo A, Deppe JL, Allen MF (2009). Using soundscape recordings to estimate bird species abundance, richness, and composition. Journal of Field Ornithology.

[b43] Cheng J, Sun Y, Ji L (2010). A call-independent and automatic acoustic system for the individual recognition of animals: a novel model using four passerines. Pattern Recognition.

[b44] Clark CW (1995). Application of U.S. Navy underwater hydrophone arrays for scientific research on whales. Report of the International Whaling Commission.

[b45] Clark CW, Borsani F, Notarbartolo-di-Sciara G (2002). Vocal activity of fin whales, *Balaenoptera physalus*, in the Ligurian Sea. Marine Mammal Science.

[b46] Clark CW, Brown MW, Corkeron P (2010). Visual and acoustic surveys for North Atlantic right whales, *Eubalaena glacialis*, in Cape Cod Bay, Massachusetts, 2001–2005: management implications. Marine Mammal Science.

[b47] Conn P, Arthur A, Bailey L, Singleton G (2006). Estimating the abundance of mouse populations of known size: promises and pitfalls of new methods. Ecological Applications.

[b48] Cox MJ, Borchers DL, Demer DA, Cutter GR, Brierley AS (2011). Estimating the density of Antarctic krill (*Euphausia superba*) from multi-beam echo-sounder observations using distance sampling methods. Applied Statistics.

[b49] Dawson DK, Efford MG (2009). Bird population density estimated from acoustic signals. Journal of Applied Ecology.

[b50] Defler T, Pintor D (1985). Censusing primates by transect in a forest of known primate density. International Journal of Primatology.

[b51] Driscoll DA (1998). Counts of calling males as estimates of population size in the endangered frogs *Geocrinia alba* and *G. Vitellina*. Journal of Herpetology.

[b52] Dudzinski KM, Brown SJ, Lammers M, Lucke K, Mann DA, Simard P, Wall CC, Rasmussen MH, Magnúsdóttir EE, Tougaard J, Eriksen N (2011). Trouble-shooting deployment and recovery options for various stationary passive acoustic monitoring devices in both shallow- and deep-water applications. The Journal of the Acoustical Society of America.

[b53] Dunn RA, Hernandez O (2009). Tracking blue whales in the eastern tropical Pacific with an ocean-bottom seismometer and hydrophone array. The Journal of the Acoustical Society of America.

[b54] Efford MG (2004). Density estimation in live-trapping studies. Oikos.

[b55] Efford MG, Dawson DK, Borchers DL (2009). Population density estimated from locations of individuals on a passive detector array. Ecology.

[b56] Falcone E, Schorr G, Douglas A, Calambokidis J, Henderson E, McKenna M, Hildebrand J, Moretti D (2009). Sighting characteristics and photo-identification of Cuvier's beaked whales (*Ziphius cavirostris*) near San Clemente Island, California: a key area for beaked whales and the military?. Marine Biology.

[b57] Figueroa H (2011). http://xbat.org/documentation.html.

[b58] Fischer FP, Schulz U, Schubert H, Knapp P, Schmoger M (1997). Quantitative assessment of grassland quality: acoustic determination of population sizes of orthopteran indicator species. Ecological Applications.

[b59] Forrest TG (1988). Using insect sounds to estimate and monitor their populations. The Florida Entomologist.

[b60] Fox E (2008). A new perspective on acoustic individual recognition in animals with limited call sharing or changing repertoires. Animal Behaviour.

[b61] Fox CG, Matsumoto H, Lau TKA (2001). Monitoring Pacific Ocean seismicity from an autonomous hydrophone array. Journal of Geophysical Research.

[b62] Fristrup KM, Clark C (1997). Combining visual and acoustic survey data to enhance density estimation. Report of the International Whaling Commission.

[b63] Gannon DP (2008). Passive acoustic techniques in fisheries science: a review and prospectus. Transactions of the American Fisheries Society.

[b64] Gardiner T, Hill J, Chesmore D (2005). Review of the methods frequently used to estimate the abundance of orthoptera in grassland ecosystems. Journal of Insect Conservation.

[b65] Gedamke J, Robinson SM (2010). Acoustic survey for marine mammal occurrence and distribution off East Antarctica (30-80°E) in January-February 2006. Deep Sea Research Part II: Topical Studies in Oceanography.

[b66] George J, Zeh J, Suydam R, Clark C (2004). Abundance and population trend (1978–2001) of western Arctic bowhead whales surveyed near Barrow. Marine Mammal Science.

[b67] Gerrodette T, Taylor BL, Swift R, Rankin S, Jaramillo-Legorreta AM, Rojas-Bracho LA (2011). Combined visual and acoustic estimate of 2008 abundance, and change in abundance since 1997, for the vaquita, *Phocoena sinus*. Marine Mammal Science.

[b68] Gillespie D, Gordon J, McHugh R, McLaren D, Mellinger DK, Redmond P, Thode A, Trinder P, Deng XY (2008). PAMGUARD: semiautomated, open source software for real-time acoustic detection and localisation of cetaceans. Proceedings of the Institute of Acoustics.

[b69] Gordon JCD (1991). Evaluation of a method for determining the length of sperm whales (*Physeter catodon*) from their vocalizations. Journal of Zoology.

[b70] Grafe TU, Meuche I (2005). Chorus tenure and estimates of population size of male European tree frogs *Hyla arborea*: implications for conservation. Amphibia-Reptilia.

[b71] Hagstrum DW, Webb JC, Vick KW (1988). Acoustical detection and estimation of *Rhyzopertha dominica* (F.) larval populations in stored wheat. The Florida Entomologist.

[b72] Harris D (2012). Estimating whale abundance using sparse hydrophone arrays.

[b73] Hastie GD, Swift RJ, Gordon JC, Slesser G, Turrell WR (2003). Sperm whale distribution and seasonal density in the Faroe Shetland Channel. Journal of Cetacean Research and Management.

[b74] Hayes SA, Mellinger DK, Croll DA, Costa DP, Borsani JF (2000). An inexpensive passive acoustic system for recording and localizing wild animal sounds. The Journal of the Acoustical Society of America.

[b75] Hiby AR, Ward AJ (1986). Analysis of cue-counting and blow rate estimation experiments carried out during the 1984/85 IDCR minke whale assessment cruise. Report of the International Whaling Commission.

[b76] Hobson KA, Rempel RS, Greenwood H, Turnbull B, Van Wilgenburg SL (2002). Acoustic surveys of birds using electronic recordings: new potential from an omnidirectional microphone system. Wildlife Bulletin.

[b77] Holt SA (2008). Distribution of red drum spawning sites identified by a towed hydrophone array. Transactions of the American Fisheries Society.

[b78] Horrocks J, Hamilton DC, Whitehead H (2011). A likelihood approach to estimating animal density from binary acoustic transects. Biometrics.

[b79] Horvitz DG, Thompson DJ (1952). A generalization of sampling without replacement from a finite universe. Journal of the American Statistical Association.

[b80] Hugel S (2012). Impact of native forest restoration on endemic crickets and katydids density in Rodrigues island. Journal of Insect Conservation.

[b81] Ichikawa K, Tsutsumi C, Arai N, Akamatsu T, Shinke T, Hara T, Adulyanukosol K (2006). Dugong (*Dugong dugon*) vocalization patterns recorded by automatic underwater sound monitoring systems. The Journal of the Acoustical Society of America.

[b82] IUCN (2003). Guidelines for Application of IUCN Red List Criteria at Regional Levels: Version 3.0.

[b83] Johnson JB, Saenz D, Burt DB, Conner RN (2009). An automated technique for monitoring nocturnal avian vocalizations. Bulletin of the Texas Ornithological Society.

[b84] Johnson MP, Tyack PL (2003). A digital acoustic recording tag for measuring the response of wild marine mammals to sound. IEEE Journal of Oceanic Engineering.

[b85] Juanes F (2002). Listening to fish: an international workshop on the application of passive acoustics in fisheries. Reviews in Fish Biology and Fisheries.

[b86] Kimura S, Akamatsu T, Li S, Dong S, Dong L, Wang K, Wang D, Arai N (2010). Density estimation of Yangtze finless porpoises using passive acoustic sensors and automated click train detection. The Journal of the Acoustical Society of America.

[b87] Kimura S, Akamatsu T, Wang K, Wang D, Li S, Dong S, Arai N (2009). Comparison of stationary acoustic monitoring and visual observation of finless porpoises. The Journal of the Acoustical Society of America.

[b88] Küsel ET, Mellinger DK, Thomas L, Marques TA, Moretti D, Ward J (2011). Cetacean population density estimation from single fixed sensors using passive acoustics. The Journal of the Acoustical Society of America.

[b89] Kyhn LA, Tougaard J, Thomas L, Duve LR, Steinback J, Amundin M, Desportes G, Teilmann J (2012). From echolocation clicks to animal density – Acoustic sampling of harbour porpoises with static dataloggers. The Journal of the Acoustical Society of America.

[b90] Laake JL, Borchers DL, Buckland ST, Anderson DR, Burnham KP, Laake JL, Borchers DL, Thomas L (2004). Methods for incomplete detection at distance zero. Advanced Distance Sampling.

[b91] Leaper R, Gillespie D, Papastavrou V (2000). Results of passive acoustic surveys for odontocetes in the Southern Ocean. Journal of Cetacean Research and Management.

[b92] Lewis T, Gillespie D, Lacey C, Matthews J, Danbolt M, Leaper R, McLanaghan R, Moscrop A (2007). Sperm whale abundance estimates from acoustic surveys of the Ionian Sea and Straits of Sicily in 2003. Journal of the Marine Biological Association of the United Kingdom.

[b93] Li S, Akamatsu T, Wang D, Wang K (2009). Localization and tracking of phonating finless porpoises using towed stereo acoustic data-loggers. The Journal of the Acoustical Society of America.

[b94] Link WA (2003). Nonidentifiability of population size from capture-recapture data with heterogeneous detection probabilities. Biometrics.

[b95] Lowe S, Skelton P (2008). First record of sound production by a South African minnow, *Pseudobarbus burchelli* (Teleostei: Cyprinidae), the Breede River redfin. African Journal of Aquatic Science.

[b96] Luczkovich JJ, Mann DA, Rountree RA (2008). Passive acoustics as a tool in fisheries science. Transactions of the American Fisheries Society.

[b97] Manly BFJ (2007). Randomization, Bootstrap and Monte Carlo Methods in Biology.

[b98] Mann DA, Hawkins AD, Jech JM, Webb JF, Fay RR, Popper AN (2010). Active and passive acoustics to locate and study fish. Fish Bioacoustics.

[b100] Marques FFC, Buckland ST (2003). Incorporating covariates into standard line transect analyses. Biometrics.

[b99] Marques TA (2004). Predicting and correcting bias caused by measurement error in line transect sampling using multiplicative error models. Biometrics.

[b101] Marques TA, Buckland ST, Borchers DL, Tosh D, McDonald RA (2010). Point transect sampling along linear features. Biometrics.

[b102] Marques TA, Munger L, Thomas L, Wiggins S, Hildebrand JA (2011). Estimating North Pacific right whale (*Eubalaena japonica*) density using passive acoustic cue counting. Endangered Species Research.

[b103] Marques TA, Thomas L, Fancy SG, Buckland ST (2007). Improving estimates of bird density using multiple covariate distance sampling. The Auk.

[b104] Marques TA, Thomas L, Martin SW, Mellinger DK, Jarvis S, Morrissey RP, Ciminello C-A, DiMarzio N (2012). Spatially explicit capture recapture methods to estimate minke whale abundance from data collected at bottom mounted hydrophones. Journal of Ornithology.

[b105] Marques TA, Thomas L, Ward J, DiMarzio N, Tyack PL (2009). Estimating cetacean population density using fixed passive acoustic sensors: an example with Blainville's beaked whales. The Journal of the Acoustical Society of America.

[b106] Martin SW, Marques TA, Thomas L, Morrissey RP, Jarvis S, DiMarzio N, Moretti D, Mellinger DK Estimating minke whale (*Balaenoptera acutorostrata*) boing sound density using passive acoustic sensors. Marine Mammal Science.

[b107] McCauley RD, Jenner CK (2010).

[b108] McClintock BT, Bailey LL, Pollock KH, Simons TR (2010). Experimental investigation of observation error in anuran call surveys. Journal of Wildlife Management.

[b109] McDonald MA (2004). DIFAR hydrophones applied to whale research. Canadian Acoustics.

[b110] McDonald MA, Fox CG (1999). Passive acoustic methods applied to fin whale population density estimation. The Journal of the Acoustical Society of America.

[b111] McDonald MA, Hildebrand JA, Wiggins SM, Johnston DW, Polovina JJ (2009). An acoustic survey of beaked whales at Cross Seamount near Hawaii. The Journal of the Acoustical Society of America.

[b112] Mellinger DK (2001).

[b113] Mellinger DK, Barlow JP (2003).

[b114] Mellinger DK, Clark CW (2000). Recognizing transient low-frequency whale sounds by spectrogram correlation. The Journal of the Acoustical Society of America.

[b115] Mellinger DK, Küsel E, Thomas L, Marques TA (2009). Taming the Jez monster: Estimating fin whale spatial density using acoustic propagation modeling. The Journal of the Acoustical Society of America.

[b116] Mellinger DK, Stafford KM, Moore SE, Dziak RP, Matsumoto H (2007). An overview of fixed passive acoustic observation methods for cetaceans. Oceanography.

[b117] Mennill DJ, Burt JM, Fristrup KM, Vehrencamp SL (2006). Accuracy of an acoustic location system for monitoring the position of duetting songbirds in tropical forest. The Journal of the Acoustical Society of America.

[b118] Moore SE, Howe BM, Stafford KM, Boyd ML (2007). Including whale call detection in standard ocean measurements: application of acoustic seagliders. Marine Technology Society Journal.

[b119] Moretti D, Marques TA, Thomas L, DiMarzio N, Dilley A, Morrissey R, McCarthy E, Ward J, Jarvis S (2010). A dive counting density estimation method for Blainville's beaked whale (*Mesoplodon densirostris*) using a bottom-mounted hydrophone field as applied to a Mid-Frequency Active (MFA) sonar operation. Applied Acoustics.

[b120] Multi-Électronique (2011). http://www.multi-electronique.com/pages/auralm2en.htm.

[b121] Munger LM, Wiggins SM, Hildebrand JA (2011). Right whale “up-call” source levels and propagation distance on the southeastern Bering Sea shelf. The Journal of the Acoustical Society of America.

[b122] Nowacek DP, Thorne LH, Johnston DW, Tyack PL (2007). Responses of cetaceans to anthropogenic noise. Mammal Review.

[b123] O'Farrell MJ, Gannon WL (1999). A comparison of acoustic versus capture techniques for the inventory of bats. Journal of Mammalogy.

[b124] Oleson EM, Calambokidis J, Barlow J, Hildebrand JA (2007). Blue whale visual and acoustic encounter rates in the Southern California Bight. Marine Mammal Science.

[b125] Parsons S, Jones G (2000). Acoustic identification of twelve species of echolocating bat by discriminant function analysis and artificial neural networks. Journal of Experimental Biology.

[b126] Parsons MJ, McCauley RD, Mackie MC, Siwabessy P, Duncan AJ (2009). Localization of individual mulloway (*Argyrosomus japonicus*) within a spawning aggregation and their behaviour throughout a diel spawning period. ICES Journal of Marine Science.

[b127] Pavan G, Adam O, Thomas L (2010). Proceedings of the 4th international workshop on detection, classification and localization of marine mammals using passive acoustics and 1st international workshop on density estimation of marine mammals using passive acoustics, University of Pavia, Collegio Cairoli, Italy, September 2009, 10–13. Applied Acoustics.

[b128] Payne KB, Thompson M, Kramer L (2003). Elephant calling patterns as indicators of group size and composition: the basis for an acoustic monitoring system. African Journal of Ecology.

[b129] Penteriani V, Gallardo M, Cazassus H (2002). Conspecific density biases passive auditory surveys. Journal of Field Ornithology.

[b130] Powell LA (2007). Approximating variance of demographic parameters using the delta method: a reference for avian biologists. The Condor.

[b131] Rankin S, Barlow J (2005). Source of the North Pacific “boing” sound attributed to minke whales. The Journal of the Acoustical Society of America.

[b132] Rebull OG, Cusí JD, Fernández MR, Muset JG (2006). Tracking fin whale calls offshore the Galicia Margin, North East Atlantic Ocean. The Journal of the Acoustical Society of America.

[b133] Risch D, Corkeron PJ, Ellison WT, Van Parijs SM (2012). Changes in humpback whale song occurrence in response to an acoustic source 200 km away. PLoS ONE.

[b134] Royle JA (2004). N-Mixture models for estimating population size from spatially replicated counts. Biometrics.

[b135] Royle JA, Link WA (2005). A general class of multinomial mixture models for anuran calling survey data. Ecology.

[b136] Royle JA, Nichols JD (2003). Estimating abundance from repeated presence–absence data or point counts. Ecology.

[b137] Royle JA, Young KV (2008). A hierarchical model for spatial capture-recapture data. Ecology.

[b138] Russo D, Jones G (2003). Use of foraging habitats by bats in a Mediterranean area determined by acoustic surveys: conservation implications. Ecography.

[b139] SCANS-II (2008). http://biology.st-andrews.ac.uk/scans2.

[b140] Schwarz CJ, Seber GAF (1999). Estimating animal abundance: review III. Statistical Science.

[b141] Seber GAF (1982). The Estimation of Animal Abundance.

[b142] Seber GAF (1986). A review of estimating animal abundance. Biometrics.

[b143] Seber GAF (1992). A review of estimating animal abundance II. International Statistical Review.

[b144] Shirose LJ, Bishop CA, Green DM, MacDonald CJ, Brooks RJ, Helferty NJ (1997). Validation tests of an amphibian call count survey technique in Ontario, Canada. Herpetologica.

[b145] Širović A, Cutter GR, Butler JL, Demer DA (2009). Rockfish sounds and their potential use for population monitoring in the Southern California Bight. ICES Journal of Marine Science.

[b146] Širović A, Hildebrand J, Wiggins S (2007). Blue and fin whale call source levels and propagation range in the Southern Ocean. The Journal of the Acoustical Society of America.

[b147] Sousa-Lima R, Paglia A, Fonseca G (2002). Signature information and individual recognition in the isolation calls of Amazonian manatees, *Trichechus inunguis* (Mammalia: Sirenia). Animal Behaviour.

[b148] Southall BL, Nowacek DP (2009). Acoustics in marine ecology: innovation in technology expands the use of sound in ocean science. Marine Ecology Progress Series.

[b149] Stafford KM, Citta JJ, Moore SE, Daher MA, George JE (2009). Environmental correlates of blue and fin whale call detections in the North Pacific Ocean from 1997 to 2002. Marine Ecology Progress Series.

[b150] Stafford K, Mellinger D, Moore S, Fox C (2007). Seasonal variability and detection range modeling of baleen whale calls in the Gulf of Alaska, 1999–2002. The Journal of the Acoustical Society of America.

[b151] Thompson ME, Schwager SJ, Payne KB (2010). Heard but not seen: an acoustic survey of the African forest elephant population at Kakum Conservation Area, Ghana. African Journal of Ecology.

[b152] Thompson ME, Schwager SJ, Payne KB, Turkalo AK (2009). Acoustic estimation of wildlife abundance: methodology for vocal mammals in forested habitats. African Journal of Ecology.

[b153] Tyack PL, Johnson M, Soto NA, Sturlese A, Madsen PT (2006). Extreme diving of beaked whales. Journal of Experimental Biology.

[b154] Van Opzeeland I, Van Parijs S, Bornemann H, Frickenhaus S, Kindermann L, Klinck H, Plötz J, Boebel O (2010). Acoustic ecology of Antarctic pinnipeds. Marine Ecology Progress Series.

[b155] Van Parijs SM, Clark CW (2006). Long-term mating tactics in an aquatic-mating pinniped, the bearded seal, *Erignathus barbatus*. Animal Behaviour.

[b156] Van Parijs SM, Clark CW, Sousa-Lima RS, Parks SE, Rankin S, Risch D, Van Opzeeland IC (2009). Management and research applications of real-time and archival passive acoustic sensors over varying temporal and spatial scales. Marine Ecology Progress Series.

[b157] Van Parijs SM, Hastie GD, Thompson PM (1999). Geographical variation in temporal and spatial vocalization patterns of male harbour seals in the mating season. Animal Behaviour.

[b158] Van Parijs SM, Smith J, Corkeron PJ (2002). Using calls to estimate the abundance of inshore dolphins: a case study with Pacific humpback dolphins *Sousa chinensis*. Journal of Applied Ecology.

[b159] Veirs V, Veirs S (2005). One year of background underwater sound levels in Haro Strait, Puget Sound. The Journal of the Acoustical Society of America.

[b160] Ward J, Jarvis S, Moretti D, Morrissey R, DiMarzio N, Thomas L, Marques TA (2011). Beaked whale (*Mesoplodon densirostris*) passive acoustic detection with increasing ambient noise. The Journal of the Acoustical Society of America.

[b161] Ward J, Morrissey R, Moretti D, DiMarzio N, Jarvis S, Johnson M, Tyack P, White C (2008). Passive acoustic detection and localization of *Mesoplodon densirostris* (Blainville's beaked whale) vocalizations using distributed bottom-mounted hydrophones in conjunction with a digital tag (DTAG) recording. Canadian Acoustics.

[b162] Ward JA, Thomas L, Jarvis S, Baggenstoss P, DiMarzio N, Moretti D, Morrissey R, Marques TA, Dunn C, Claridge D, Hartvig E, Tyack P (2012). Passive acoustic density estimation of sperm whales in the Tongue of the Ocean, Bahamas. Marine Mammal Science.

[b163] Wentz GM, Tavolga WN (1964). Curious noises and the sonic environment in the ocean. Marine Bio-Acoustics.

[b164] Whitehead H (2009). Estimating abundance from one-dimensional passive acoustic surveys. Journal of Wildlife Management.

[b165] Wiggins S (2003). Autonomous Acoustic Recording Packages (ARPs) for long-term monitoring of whale sounds. Marine Technology Society Journal.

[b166] Wiggins SM, McDonald MA, Hildebrand JA (2012). Beaked whale and dolphin tracking using a multichannel autonomous acoustic recorder. The Journal of the Acoustical Society of America.

[b167] Wildlife Acoustics, Inc (2012). http://www.wildlifeacoustics.com/products/acoustic-monitoringand…/marine-monitoring.

[b168] Willcox S, Manley J, Wiggins S (2009). The Wave Glider, an energy harvesting autonomous surface vessel. Sea Technology.

[b169] Williams BK, Nichols JD, Conroy MJ (2002). Analysis and Management of Animal Populations.

[b170] Wood JD (2010). Marine mammal species conservation: a review of developments in the uses of acoustics. Journal of International Wildlife Law and Policy.

[b171] Zhang M, Crocker RL, Mankin RW, Flanders KL, Hubbard JLB (2003). Acoustic estimation of infestations and population densities of white grubs (Coleoptera: Scarabaeidae) in turfgrass. Journal of Economic Entomology.

[b172] Zimmer WMX (2011). Passive Acoustic Monitoring of Cetaceans.

[b173] Zimmer WM, Harwood J, Tyack PL, Johnson MP, Madsen PT (2008). Passive acoustic detection of deep diving beaked whales. The Journal of the Acoustical Society of America.

[b174] Zwolinski J, Fernandes PG, Marques V, Stratoudakis Y (2009). Estimating fish abundance from acoustic surveys: calculating variance due to acoustic backscatter and length distribution error. Canadian Journal of Fisheries and Aquatic Sciences.

